# Genomic diversity, linkage disequilibrium and selection signatures in European local pig breeds assessed with a high density SNP chip

**DOI:** 10.1038/s41598-019-49830-6

**Published:** 2019-09-19

**Authors:** M. Muñoz, R. Bozzi, J. García-Casco, Y. Núñez, A. Ribani, O. Franci, F. García, M. Škrlep, G. Schiavo, S. Bovo, V. J. Utzeri, R. Charneca, J. M. Martins, R. Quintanilla, J. Tibau, V. Margeta, I. Djurkin-Kušec, M. J. Mercat, J. Riquet, J. Estellé, C. Zimmer, V. Razmaite, J. P. Araujo, Č. Radović, R. Savić, D. Karolyi, M. Gallo, M. Čandek-Potokar, A. I. Fernández, L. Fontanesi, C. Óvilo

**Affiliations:** 1 Departamento Mejora Genética Animal, INIA, Madrid, Spain; 2 DAGRI, Animal Science Section, Università degli Studi di Firenze, Firenze, Italy; 3 Department of Agricultural and Food Sciences, University of Bologna, Bologna, Italy; 4 Kmetijski inštitut Slovenije, Hacquetova ulica 17, SI-1000 Ljubljana, Slovenia; 5 Instituto de Ciências Agrárias e Ambientais Mediterrânicas (ICAAM), Universidade de Évora, Évora, Portugal; 6 IRTA, Programa de Genética y Mejora Animal, Barcelona, Spain; 7 Faculty of Agrobiotechnical Sciences Osijek, University of Osijek, Osijek, Croatia; 8 IFIP – Institut du Porc, Le Rheu, France; 9 INRA, Génétique Physiologie et Système d’Elevage, Castanet-Tolosan, France; 10 GABI, INRA, AgroParisTech, Université Paris-Saclay, Jouy-en-Josas, France; 11 Bäuerliche Erzeugergemeinschaft Schwäbisch Hall, Wolpertshausen, Germany; 12 Animal Science Institute, Lithuanian University of Health Sciences, Baisogala, Lithuania; 13 Centro de Investigação de Montanha (CIMO), Instituto Politécnico de Viana do Castelo, Escola Superior Agrária, Ponte de Lima, Portugal; 14 Institute for Animal Husbandry-Pig Research Department, Autoput for Zagreb 16, 11080 Belgrade-Zemun, Serbia; 15 University of Belgrade, Faculty of agriculture, Nemanjina 6, 11080 Belgrade-Zemun, Serbia; 16 Department of Animal Science, University of Zagreb, Faculty of Agriculture, Zagreb, Croatia; 17 Associazione Nazionale Allevatori Suini (ANAS), Roma, Italy

**Keywords:** Animal breeding, Genomics, Population genetics

## Abstract

Genetic characterization of local breeds is essential to preserve their genomic variability, to advance conservation policies and to contribute to their promotion and sustainability. Genomic diversity of twenty European local pig breeds and a small sample of Spanish wild pigs was assessed using high density SNP chips. A total of 992 DNA samples were analyzed with the GeneSeek Genomic Profiler (GGP) 70 K HD porcine genotyping chip. Genotype data was employed to compute genetic diversity, population differentiation and structure, genetic distances, linkage disequilibrium and effective population size. Our results point out several breeds, such as Turopolje, Apulo Calabrese, Casertana, Mora Romagnola and Lithuanian indigenous wattle, having the lowest genetic diversity, supported by low heterozygosity and very small effective population size, demonstrating the need of enhanced conservation strategies. Principal components analysis showed the clustering of the individuals of the same breed, with few breeds being clearly isolated from the rest. Several breeds were partially overlapped, suggesting genetic closeness, which was particularly marked in the case of Iberian and Alentejana breeds. Spanish wild boar was also narrowly related to other western populations, in agreement with recurrent admixture between wild and domestic animals. We also searched across the genome for loci under diversifying selection based on F_ST_ outlier tests. Candidate genes that may underlie differences in adaptation to specific environments and productive systems and phenotypic traits were detected in potentially selected genomic regions.

## Introduction

Pork is the most widely consumed meat worldwide^1^. Pig industry is mainly based on a limited number of cosmopolitan lean breeds which have been extensively used for breeding improvement. These highly selected breeds are raised in intensive production systems, focused on maximizing productivity, and supplying the market with fresh pork. Besides, many local, less performing breeds exist, although some of them are nowadays close to extinction. These traditional breeds are usually associated with local forms of pig husbandry and their meat is used for the production of high-quality and niche products. The Iberian pig, raised in the South-West of the Iberian Peninsula, is probably the most representative local pig breed, although many others are reared in European countries. Common characteristics of these breeds are a good environmental adaptation, rusticity, low muscle mass deposition and high adipogenic potential and, in many cases, superior meat quality traits^2^.

Conservation of small local breeds is mainly conditioned by their economic relevance but also depends on socio-cultural value, adaptation to local agro-climatic conditions, contribution to the development of local communities and marginal areas, and scientific importance. Efficient utilization of local breeds is needed and for this goal, technological advances, growing knowledge and innovative ideas founded on scientific research must be developed. The genetic characterization of these resources is a preliminary step for the development of conservation programs and to boost local breed promotion, and their sustainable use^3^.

Despite a few whole genome studies have been carried out for some European local pig breeds, such as the Iberian^4,5^ or Casertana^6,7^, many other autochthonous breeds have not been analyzed in detail yet and are considered untapped genetic resources. Recently, the diversity at several relevant candidate gene polymorphisms has been evaluated in twenty local European pig breeds^8^, providing information about the segregation of interesting markers for breeding or traceability^9,10^ purposes and giving some first insights into the genetic structure of these populations. Nevertheless, genetic characterization of animal breeds is usually addressed with the analysis of neutral markers like microsatellites or SNPs, as recommended by FAO^11^. High density SNP panels help to investigate genome wide diversity with a higher resolution. Dense SNP panels can be applied to a variety of genomics studies including inference on population history, structure and admixture^12^, estimation of effective population size^4,5^, QTL mapping strategies^13^ and whole genome association studies^14^ and genomic selection^15^. Also, comparative genomic diversity enables us to explore the degree of genomic variation and linkage disequilibrium (LD) among pig breeds. This as well helps to detect genomic regions that have been subject to selective sweeps in different pig populations^16^.

In this study, we analyzed genomic diversity of 20 European local pig breeds: Black Slavonian and Turopolje (Croatia), Basque and Gascon (France), Schwäbisch-Hällisches Schwein (Germany), Apulo-Calabrese, Casertana, Cinta Senese, Mora Romagnola, Nero Siciliano and Sarda (Italy), Lithuanian indigenous wattle and Lithuanian White old type (Lithuania), Alentejana and Bísara (Portugal), Moravka and Swallow-Bellied Mangalitsa (Serbia), Krškopolje pig (Slovenia) and Iberian and Majorcan Black (Spain). The genotyping information from a high density SNP chip has been employed to assess genomic diversity and structure and to identify selection signatures. This work is framed within the TREASURE project (https://treasure.kis.si), a multidisciplinary European Union funded project pointing toward the development of sustainable pork chains in several European local pig breeds.

## Results and Discussion

**Figure 1:**
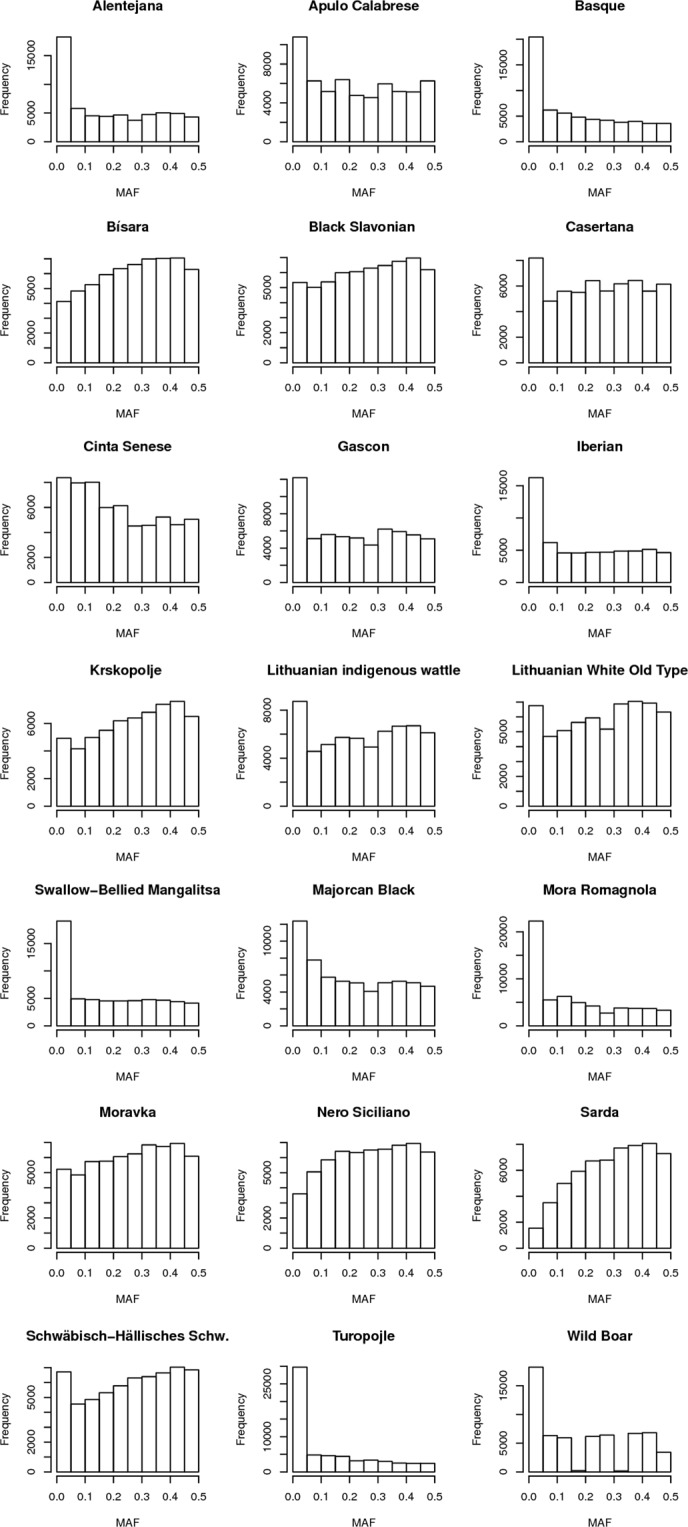
Frequency distribution of minor allele frequencies (MAF) in all the breeds.

A total of 985 pigs from 20 European autochthonous breeds and a small Spanish Wild Boar population (n = 7) were successfully genotyped with the GeneSeek ® Genomic Profiler (GGP) 70 K HD Porcine chip (Illumina Inc, USA). The sample sizes of each analyzed population are included in Table 1. Selection of animals for sampling was performed by local specialized personnel with a deep knowledge of each breed, in order to get representative samples within each analyzed population. A total of 60,451, out of 68,516 SNPs, remained after the removal of those with more than 10% missing genotypes or Minor Allele Frequency (MAF) lower than 0.01. The average within-breed MAF (Table 1, Fig. 1) ranged from 0.133 (Turopolje) to 0.294 (Sarda). In agreement with these results, Turopolje was the breed with the highest number of SNPs (29,740) with the lowest MAF values (ranging between 0.01 and 0.05) while Sarda had the highest number (15,350) of highly informative SNP markers with frequencies between 0.4 and 0.5 (Fig. 1 and Supplementary Table 1). The breeds showing the lowest number of informative SNPs were Alentejana, Basque, Iberian, Swallow-Bellied Mangalitsa, Mora Romagnola, Turopolje and Wild Boar, since more than 25% of their SNPs had MAF values lower than 0.05. On the contrary, the breeds showing the most informative genotyping results, with more than 20% of their SNPs with MAF higher than 0.40 were Sarda, Krškopolje, Schwäbisch-Hällisches Schwein, Bísara, Nero Siciliano, Old type Lithuanian White, Black Slavonian, Moravka and Lithuanian indigenous wattle.

**Table 1:** Samples sizes (N), mean minor allele frequencies (MAF), observed (H_O_) and expected (H_E_) heterozigosities, inbreeding coefficient of an individual (I) relative to the subpopulation (S) (F_IS_) and Wright’s fixation index (F_ST_), for each analyzed breed.

Breed	N	MAF	H_O_	H_E_	F_IS_	F_ST_
Alentejana	48	0.193	0.248	0.259	0.041	0.116
Apulo Calabrese	53	0.228	0.258	0.305	0.138	0.118
Basque	39	0.169	0.240	0.233	−0.026	0.147
Bísara	49	0.270	0.339	0.355	0.045	0.102
Black Slavonian	49	0.262	0.332	0.346	0.040	0.096
Casertana	54	0.246	0.291	0.327	0.095	0.110
Cinta Senese	54	0.220	0.300	0.300	0.011	0.101
Gascon	48	0.224	0.299	0.298	−0.005	0.122
Iberian	49	0.202	0.251	0.270	0.077	0.110
Krškopolje pig	52	0.277	0.363	0.361	−0.003	0.109
Lithuanian indigenous wattle	48	0.249	0.354	0.331	−0.066	0.116
Majorcan Black	48	0.210	0.279	0.285	0.005	0.102
Swallow-Bellied Mangalitsa	50	0.192	0.257	0.259	0.006	0.125
Mora Romagnola	48	0.161	0.230	0.220	−0.039	0.161
Moravka	50	0.267	0.348	0.353	0.014	0.101
Nero Siciliano	50	0.272	0.341	0.360	0.052	0.085
Lithuanian White Old Type	51	0.260	0.358	0.341	−0.049	0.119
Sarda	48	0.294	0.358	0.382	0.060	0.092
Schwäbisch-Hällisches Schwein	49	0.264	0.349	0.342	−0.016	0.110
Turopolje	50	0.133	0.195	0.187	0.046	0.159
Wild Boar	7	0.192	0.240	0.254	0.041	0.132
Average	47	0.228 (0.044)	0.297 (0.053)	0.303 (0.054)	0.022 (0.050)	0.115 (0.020)

In general, the informativity of the SNP chip was moderate in most of the studied breeds, which is not unexpected as these local breeds have not been considered for the design of commercial porcine chips^17^. The most widely used Illumina 60 K chip was designed and validated using samples from Berkshire, Duroc, Hampshire, Landrace, Large White, Meishan, Pietrain, Synthetic lines (Large White and Piétrain) and wild boar^17^. The GeneSeek ® GGP Porcine HD Genomic Profiler used in the present study is an improved version of the latter one, which was designed to include the most informative SNPs from the Porcine60K chip, according to findings in the same major commercial breeds, and that was complemented with new SNPs to improve the coverage of the chromosomes and the telomere regions, to better account for recombination^18^. In spite of the ascertainment bias implicit in the use of SNP chips, previous results have shown that these tools provide reliable estimates of genomic diversity for comparative studies between European populations, even in local breeds^19^.

### Genetic diversity parameters and population structure

#### Within-breed genetic diversity

Genetic variability parameters for the analysed populations are presented in Table 1. Within-breed observed (H_O_) and expected (H_E_) heterozygosity ranged from 0.195 (Turopolje) to 0.363 (Krškopolje) and from 0.187 (Turopolje) to 0.382 (Sarda), respectively (Table 1). Turopolje, Mora Romagnola, Basque and Wild Boar exhibit the lowest H_O_ and H_E_ values and Krškopolje, Sarda and Old type Lithuanian White, the highest ones. Across-breeds averaged values for H_O_ and H_E_ were 0.297 (±0.053) and 0.303 (±0.054), respectively. These heterozygosity values are considerably lower than those reported previously for European cosmopolitan and Chinese pig breeds^20–23^, which ranged from 0.30–0.40 to 0.60–0.70 with an average of about 0.5, and similar to those reported for some local breeds^19^. The lowest heterozygosity values observed in Turopolje are in agreement with a previous study in which microsatellites were employed to assess the genetic diversity and population structure in eight populations corresponding to Balkan pig breeds^24^. In addition, Lithuanian indigenous wattle shows the smallest (−0.066) inbreeding coefficient of an individual relative to the subpopulation (F_IS_) while Apulo Calabrese showed the highest one (0.138) (Table 1). Negative F_IS_ values were observed in Mora Romagnola, Schwäbisch-Hällisches Schwein, the two Lithuanian and the two French breeds (Gascon and Basque). Negative values indicate random mating among the individuals of the subpopulations but do not necessarily imply lower values of the total inbreeding coefficient which takes into account the accumulated inbreeding along the generations^25^.

Lack of selection programs and frequent or recurrent admixture, common to these untapped local pig breeds, should presumably lead to a higher degree of genetic diversity. However, the level of genetic variation in our local breeds is in general lower than that in cosmopolitan pig breeds^16–20^. The reason for this could be due to the small effective population size: in some of the breeds, only few founders were left at the beginning of the preservation programs^2^. This can also be the cause for high level of inbreeding observed in some of these breeds. For instance, the highest F_IS_ values obtained in Apulo Calabrese and Casertana are in agreement with their endangered situation and small census^26,27^ and similar values were reported recently in an analysis based on candidate gene polymorphisms^8^.

#### Differentiation among breeds and genetic distances

The level of population differentiation can be quantified using the fixation indexes. The fixation Index (F_ST_) for each breed (Table 1), estimated as the average of breed pairwise comparisons per SNP and then averaged by breed, shows the highest value for Mora Romagnola (0.161) and the lowest value for Sarda (0.092). The overall F_ST_ value from all the SNP markers was 0.115 (±0.020), indicating that most of the genetic variation occurred within populations rather than between breeds, as previously reported for pig populations^28,29^. The value obtained is concordant with previous works^28^, where the averaged F_ST_ value calculated between European breeds was 0.134, ranging from 0.021 to 0.209. The overall F_ST_ value obtained in the present work (analyzing data of 70K SNP chip) is considerably lower than the one observed in our previous study (0.27)^8^ performed with an array of selected SNPs. This difference is expected because in the latter study only 39 causal mutations and polymorphisms in candidate genes were used whereas, in the present work, SNP markers across the whole genome and mostly neutral were used^6^. In addition to this, the distribution of different genetic diversity parameters estimated for each SNP marker is shown in Supplementary Fig. 1.

**Figure 2:**
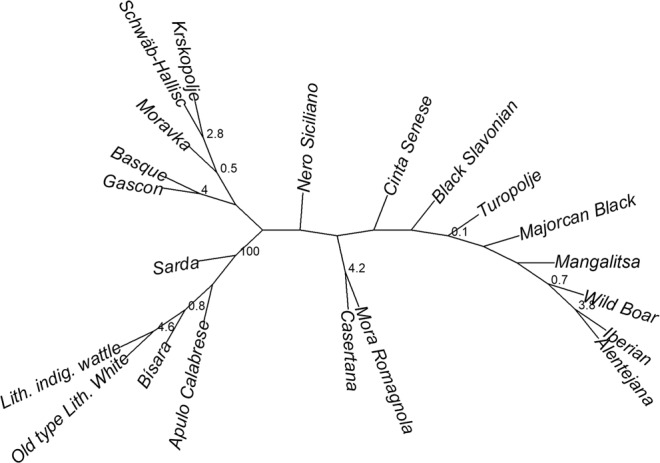
Neighbor-joining tree constructed with Nei’s distances. Numbers correspond to the percentage in which the node is recovered.

Nei’s genetic distances^30^ between studied breeds range from the minimum value of 0.276 (observed between Alentejana and Iberian breeds) to the maximum value of 0.604 (observed between Apulo Calabrese and Mora Romagnola) (Supplementary Table 2), with an averaged genetic distance of 0.440 (±0.057). The Neighbor-Joining tree (NJ) constructed from these genetic distances (Fig. 2) is in general agreement with the geographical distribution of most of these breeds, namely the breeds geographically close cluster together, such as the two French breeds; Iberian, Alentejana and Wild Boar which come from the Iberian Peninsula; the two Lithuanian breeds (Lithuanian indigenous wattle and Lithuanian White old type), and the six Italian breeds (Apulo Calabrese, Casertana, Cinta Senese, Mora Romagnola, Nero Siciliano and Sarda) that are all placed in the middle of the unrooted tree (Fig. 2). These findings are expected considering that closely located breeds are more likely to share common ancestors. Besides this resemblance with the geographical distribution of the breeds, this tree cannot be used to infer any phylogenetic relationships, considering the complexity of events that might have contributed to construct the current genetic pools of the investigated breeds which share a common European origin.

**Figure 3:**
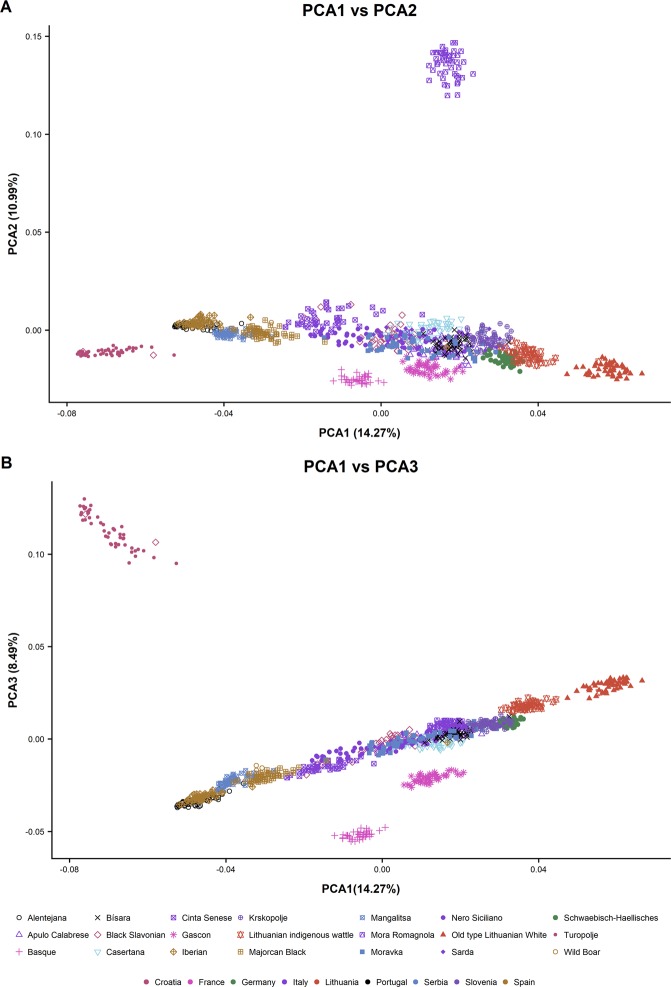
Genetic structure of the investigated 20 porcine breeds and Wild Boar population. Each point represents the eigenvalues of principal components 1 and 2 (**A**) and 2 and 3 (**B**). Points are colored according to the country and the shapes represent the different breeds.

Principal component analysis (PCA) was employed to explore the clustering of individuals of different populations. The first three principal components explained 14.26%, 10.92% and 8.49% of the total variation. PCA allowed the visualization of groups formed by individuals belonging to the same breeds (Fig. 3). Moreover, clearly separated clusters were observed for Mora Romagnola, Turopolje, Gascon, Basque and Old Type Lithuanian White breeds. Some relationship of the clusters with the breeds’ geographical distribution could also be distinguished in few cases. For instance, Alentejana and Spanish populations group together as well as French and Lithuanian breeds, in agreement with the constructed NJ tree. On the other hand, the two Croatian breeds (Turopolje and Black Slavonian) plot very distant to each other, although two Black Slavonian pigs cluster with the isolated Turopolje group. The net differentiation between the two Croatian breeds is in agreement with previous results^24^ obtained after the study of several Balkan breeds with microsatellite markers, showing a clear distinction which is now confirmed at the genomic level and with a wider panel of breeds. The isolation of Turopolje breed is not surprising, as this breed is among the oldest ones in Europe, apparently being locally domesticated in the Middle Ages^31^, while Black Slavonian breed was formed at the end of the 19th century by crossing Mangalitsa with several imported breeds of pig. However, as both breeds were raised in the past in the same geographical area, the observed few exceptions in clustering may result from uncontrolled mating between them^32^. Moreover, previous works using microsatellite markers already showed higher genetic heterogeneity in the Black Slavonian population with one herd being clustered together with Turopolje pigs, suggesting moderate gene flow between the Black Slavonian breed and the Turopolje population, matching our results^20^.

Although the individuals are grouped by breeds, there is some overlapping among them, with a big cluster including many breeds. In the left end of this big cluster, Iberian and Alentejana breeds are completely overlapped, in agreement with their genetic closeness and common breeding history^2,8^. Next to the Iberian cluster, Spanish Wild Boar and Majorcan Black pigs are also closely grouped, all composing a nucleus of South-Western European populations. A close relatedness between the domestic breeds and the wild relative is observed. This finding is in agreement with the previously proposed recurrent admixture between wild and domesticated animals in Europe^10,33^, which might be especially intense in these local breeds, exploited in free-range systems, favoring a long history of genetic exchange with wild boars. The only breed coming from the Iberian Peninsula and being located far away from the south-western cluster is the Bísara breed, for which the separation from Iberian breed had been previously reported^8,12^. This is in agreement with its Celtic origin^34^. Interestingly (Swallow-bellied) Mangalitsa pigs are located in the middle of the western nucleus, quite far away from Moravka (the other Serbian breed), as already evidenced with the NJ tree. Genetic proximity between Hungarian Mangalitsa and Iberian pigs was reported previously by Herrero-Medrano *et al*.^19^. Next, four Italian breeds (all but Mora Romagnola) are clustered in the middle, together with Moravka and Black Slavonian pigs. The right end of the Italian cluster partially overlaps with Bísara and Krškopolje breeds, which are followed by Schwäbisch-Hällisches Schwein and Lithuanian Indigenous wattle, which is in the right end, close but separated from the other Lithuanian breed.

Interestingly, the breeds located in the right end of the PCA plots (Fig. 3A,B) are those with the highest heterozygosity values, which may be related to introgression or admixture with other breeds. In some cases Asian introgression, common in many European breeds, may be the explanation. In fact, for instance, the Old type Lithuanian White breed was developed by improving Lithuanian Indigenous wattle pigs with Large White, Middle White, Edelschwein, Berkshire and local Danish pigs^35^, which may explain its apparently high genetic diversity, despite several bottlenecks occurred since 2003, leading to a critical situation nowadays. The Schwäbisch-Hällisches Schwein breed was originated by crossing local pigs in Württemberg with Chinese pigs, and later by crossbreeding with pigs imported from England^2^. The creation of Black Slavonian breed, which was a dominant one on the territory of Croatia up to the mid-20th century, resulted from planned crossing between Mangalitsa, Berkshire, Poland China and Large Black (Cornwall) pig^36,37^. On the other hand, several breeds have been subjected to crossbreeding with cosmopolitan European breeds carrying Asiatic introgression, such as Krškopolje, which was likely crossed with German Landrace in times when the breed was cast out. Regarding the two Serbian breeds, Moravka was created as a result of unsystematic crossings of the old pig Šumadinka with Berkshire and possibly with Yorkshire^38^, while no Asian introgression is known for Swallow-Bellied Mangalitsa. This may explain the separation of both breeds observed in the NJ tree and PCA plots, as well as the proximity of Mangalitsa breed to the south-western populations, which are free of Asiatic introgression.

#### Linkage disequilibrium (LD) analyses

Differences in LD among populations result from the differences in population history and demography^39,40^ and detailed information on LD in domesticated animals is important because it is of high utility for fine mapping of genes^41^. Usually, a substantial extent of LD has been found in domestic species, which may be due to small effective population size in commercial populations. Our study provides an overview of LD patterns against physical distance in 20 European pig breeds and Iberian Wild Boar.

Different SNP marker sets defined by breed (Supplementary Table 3) were used to estimate LD for all SNP pairs in a distance lower than 50 Mb, dividing this window into three different categories: (a) 0 to 2 Mb, (b) 2 to 5 Mb, (c) 5 to 50 Mb; and averaging the r^2^ values in distance bins of 0.05, 0.20 and 5.0 Mb for classes a), b) and c), respectively (Supplementary Table 4). Overall LD across the genome between adjacent SNPs ranged from 0.289 in Wild Boar to 0.604 in Mora Romagnola.

**Figure 4:**
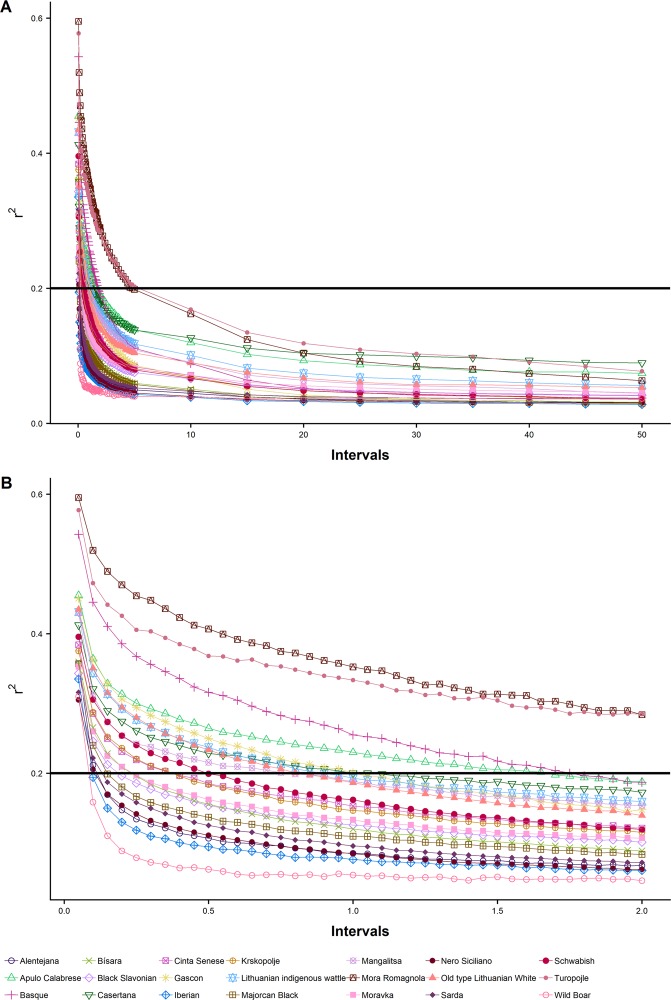
Linkage disequilibrium decay. Average linkage disequilibrium plotted against distance between SNPs across the 18 autosomes for each breed.

Overall r^2^ values by breed were plotted against increasing distances (Fig. 4). As expected, most tightly linked SNP pairs have the highest r^2^ and average r^2^ rapidly decreases as distance increases, with a similar pattern to what has been observed in previous studies in pigs and in other species^42–45^. Values of r^2^ at short distances (0.00–0.05 Mb) ranged from 0.305 (Nero Siciliano) to 0.595 (Mora Romagnola); and at long distances (45–50 Mb) r^2^ values ranged from 0.028 (Iberian) to 0.089 (Casertana). The persistence and strength of LD varied among breeds. Focusing on the domestic breeds, while the LD of Iberian and Alentejana breeds decreased by the half at 0.15 Mb, showing the highest LD decay, the LD of Mora Romagnola and Turopolje decreased by the half at 1.8 and 1.75 Mb, respectively, showing the highest LD persistence. In addition to this, Fig. 4 reveals that all breeds showed r^2^ < 0.2 at distances lower than 2 Mb except Mora Romagnola and Turopolje, which showed r^2^ < 0.2 at distances lower than 5 Mb. This high level of long LD extent could point out that these breeds have experienced more unbalanced contributions (bottlenecks) or genetic drift compared with the other ones^40^. Similar r^2^ values for all the distances were observed for Iberian and Alentejana breeds, supporting the genetic closeness of these breeds already described above. Wild Boar showed the lowest extent of LD in agreement with previous findings^28^ and as expected for an outbred and non-admixed population. In a previous work^44^, European pig breeds showed high levels of significant differences in the extent of LD, in agreement with our results.

Pairwise r^2^ estimates for short distances (0.05 Mb) were averaged by autosomes in all the breeds (Supplementary Table 5). These estimates revealed variation among chromosomes. While SSC1 was the chromosome with the highest LD in most breeds (Alentejana, Basque, Bísara, Casertana, Iberian, Krškopolje, Lithuanian indigenous wattle, Majorcan Black, Swallow-Bellied Mangalitsa, Moravka, Nero Siciliano, Old type Lithuanian White, Sarda, Schwäbisch-Hällisches Schwein, Wild Boar), SSC10 was the chromosome with the lowest LD observed in many breeds (Alentejana, Basque, Bísara, Black Slavonian, Cinta Senese, Iberian, Krškopolje, Swallow-Bellied Mangalitsa, Moravka, Nero Siciliano, Old type Lithuanian White, Schwäbisch-Hällisches Schwein, Turopolje, Wild Boar). Chromosomes with highest and lowest LD mean values were SSC3 (Mora Romagnola, r^2^ = 0.728) and SSC10 (Nero Siciliano, r^2^ = 0.256), respectively.

#### Effective population size across generations

**Figure 5:**
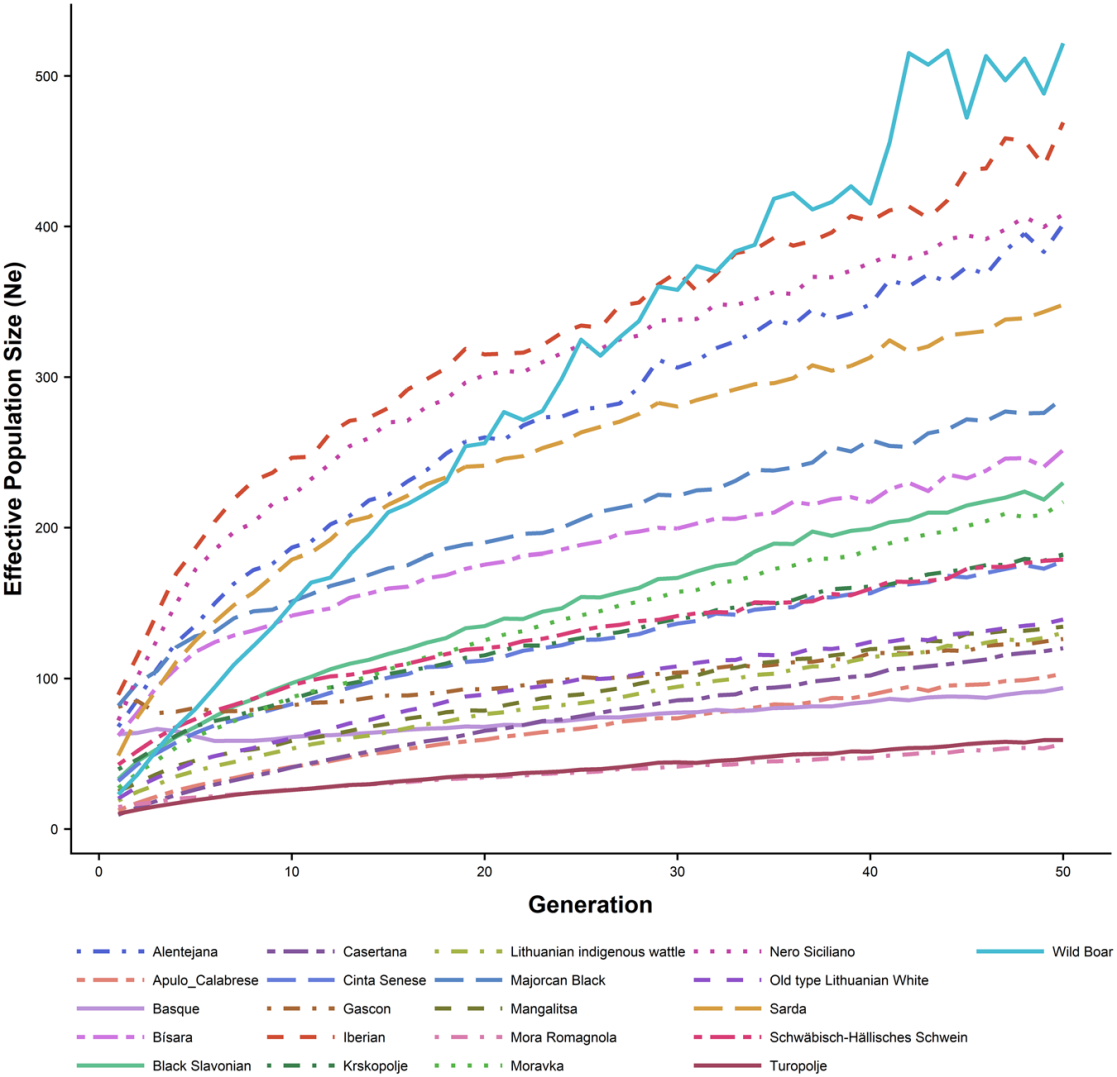
Estimated effective population size (Ne) along 50 generations.

Synteny r^2^ estimates between all pairs within 50 Mb were computed to estimate N_e_ across 50 generations (Fig. 5, Supplementary Tables 6–26) using the recombination values for each chromosome showed in Supplementary Table 3. Wild Boar had the highest N_e_ 50 generations ago (521.68) and Mora Romagnola and Turopolje were the breeds with the lowest Ne values (56.63 and 59.08, respectively). Table 2 shows the current effective population size, with Iberian pig having the highest N_e_ (89.18), and Casertana the lowest value (9.44).

**Table 2:** Current effective population size (Ne), standard deviation (SD) between brackets and sample size (N) by breed.

Breed	Ne (SD)	N
Alentejana	67.96 (0.10)	48
Apulo Calabrese	12.38 (0.01)	53
Basque	62.98 (0.12)	39
Bísara	62.01 (0.06)	49
Black Slavonian	33.11 (0.03)	49
Casertana	9.44 (0.01)	54
Cinta Senese	31.82 (0.03)	54
Gascon	81.12 (0.12)	48
Iberian	89.18 (0.16)	49
Krškopolje pig	39.63 (0.03)	52
Lithuanian indigenous wattle	18.81 (0.01)	48
Majorcan Black	81.86 (0.12)	48
Swallow-Bellied Mangalitsa	25.15 (0.02)	50
Mora Romagnola	14.68 (0.01)	48
Moravka	27.25 (0.02)	50
Nero Siciliano	72.14 (0.08)	50
Old type Lithuanian White	20.30 (0.01)	51
Sarda	48.81 (0.04)	48
Schwäbisch-Hällisches Schwein	42.74 (0.04)	49
Turopolje	10.21 (0.01)	50
Wild Boar	23.01 (0.06)	7

Meuwissen^46^ recommended an effective population size of 100 in order to maintain the genetic diversity of a population, which is not accomplished in any of the populations analysed in the present work. Our findings further confirm the need for conservation strategies for many of the studied breeds. The most extreme cases are Casertana, Apulo Calabrese, Turopolje, Mora Romagnola and both Lithuanian pig breeds, for which conservation efforts are currently being undertaken^27,47–49^.

In fact, most local breeds are characterized by having small effective population sizes, which affects their diversity, and leads to high levels of LD and a high proportion of SNPs with fixed alleles. In general, breeds showing the highest levels of LD were those with the lowest effective population size and higher inbreeding (Apulo Calabrese, Casertana and Turopolje).

#### F_ST_ analyses

The estimation of F_ST_ index was used in order to detect genomic regions that could be involved in domestication, breed pattern establishment or selective breeding. A genome-wide scan of divergent genomic regions was carried out through the estimation of Wright’s F_ST_ at each marker as a measure of genetic differentiation. Candidate regions to diversifying selection were identified as those in the 99^th^ percentile of the empirical distributions of sliding windows (Supplementary Figs 2–22). A total of 502 windows per breed were identified as outlier windows (Supplementary Tables 27–47) and when the outlier windows were adjacent, they were considered as the same genomic regions.

A total of 19 genomic regions overlapped in five or more pig groups (breeds and/or wild boars) on chromosomes SSC1, SSC2, SSC6, SSC7, SSC8 and SSC13 (Table 3). In these regions, candidate genes related with reproduction (*ADAD1*, *PRDM1 SPACA1* and *SLCO4C1*), lipid, carbohydrate and protein metabolism (*PGD*, *UBE4B*, *RNF150*, *UBE2E1*, *CNR1*, *RBP7* and *STARD4*), growth and development (*FER*, *IL2*, *IL15*, *IL21 and PRDM1*), cellular homeostasis (*ATG5*), locomotor behavior (*NOVA1*, *SOBP*) and response to nutrient (*BCKDHB*) were identified. The region located on SSC8 (100.93–101.74) overlapped in seven breeds (Alentejana, Apulo Calabrese, Bísara, Cinta Senese, Gascon, Krškopolje, Moravka) and in Wild Boar and contained genes involved in growth and reproduction. The breeds with the highest number of overlapped regions (with five or more breeds) were Alentejana and Iberian in addition to Wild Boar (12 regions), whereas the breed with the lowest number was Mora Romagnola, in agreement with a specific genetic differentiation in this breed, also highlighted in the PCA analyses.

**Table 3:** Genomic regions with outlier FST-windows shared among at least five breeds and genes annotated within these regions in Sscrofa11.1.

Chr	Position (Mb)	Breeds	Genes	Function
SSC1	55.71–56.47	AL, AC, CS,LI,MB	*Sperm Acrosome Associated 1* (*SPACA1*)	Spermatogenesis
			*Cannabinoid Receptor 1* (*CNR1*)	Intramuscular fat deposition
SSC1	63.51–63.94	AL, IB, KR, LI, MB, NS	—	—
SSC1	70.86–74.09	AL, BI, IB, KR, LI,OW, SW	*PR/SET Domain 1* (*PRDM1*)	Embrionic development
			*Autophagy Related 5* (*ATG5*)	Autophagy; cellular homeostasis
			*Sine Oculis Binding Protein Homolog* (*SOBP*)	Locomotory Behaviour. Sensory perception
SSC1	84.00–85.65	AL, BA, IB, MB, SW	*Branched Chain Keto Acid Dehydrogenase E1 Subunit Beta* (*BCKDHB*)	Response to nutrient
SSC2	107.46–108.09	AL, CS, IB, SA, TU, WB	*Solute Carrier Organic Anion Transporter Family Member 4C1* (*SLCO4C1*)	Establishment and maintenance of pregnancy
SSC2	111.79–112.28	IB, MA, MV, TU, WB	—	—
SSC2	112.94–114.00	AL, BI, IB, MA, WB	*FER Tyrosine Kinase* (*FER*)	Cell proliferation; Growth factor
SSC2	115.83–116.31	AL, CA, LI, MA, WB	*Star Related Lipid Transfer Domain Containing 4* (*STARD4*)	Cholesterol metabolism
SSC6	23.81–24.40	AL, IB, MB, SA, WB	—	—
SSC6	65.52–66.05	BI, BS, GA, KR, SA, WB	adherens junctions associated protein 1 (*AJAP1*)	Cell adhesion
SSC6	70.29–70.71	AC, BA, BS, CA,KR, NS, WB	*Retinol Binding Protein 7* (*RBP7*) ubiquitination factor E4B (*UBE4B*) kinesin family member 1B (*KIF1B*) phosphogluconate dehydrogenase (*PGD*)	Lipid binding Protein ubiquitination Vesicle transport. Development of nervous system Carbohydrate metabolism
SSC7	71.73–73.13	CA, GA, MB, MV, OW	NOVA alternative splicing regulator 1 (*NOVA1*)	Locomotory behaviour
SSC8	57.90–60.06	BA, CA, CS, GA, MB, NS	—	—
SSC8	85.68–86.25	AL, BA, BI, GA, IB, MR, MV	*Interleukin 15* (*IL15*) Ring finger protein 150 (*RNF150*)	Cell maturation Protein ubiquitination
SSC8	92.95–96.07	AL, BA, CA, CS,GA, WB	Sodium channel and clathrin linker 1 (*SCLT1*)	Ciliogenesis
SSC8	99.32–99.73	AL, AC, GA, MV, SA, WB	—	—
SSC8	100.93–101.74	AL, AC, BI, CS, GA, KR, MV, WB	*Interleukin 21* (*IL21*)	Growth factor activity
			*Interleukin 2* (*IL2*)	Growth factor activity
			*Adenosine Deaminase Domain Containing 1* (*ADAD1*)	Spermatid development
SSC13	10.52–10.63	CS, IB, KR, MA, SA, WB	Ubiquitin conjugating enzyme E2 E1 (UBE2E1)	Protein ubiquitination
SSC13	14.11–15.16	IB, MA, MB, NE, SA,TU, WB	Eomesodermin *(EOMES)* 5-azacytidine induced 2 (AZI2)	Embryonic development Immunity

AL: Alentejana; AC: Apulo Calabrese; BA: Basque; BI: Bísara; BS: Black Slavonian; CA: Casertana; CS: Cinta Senese; GA: Gascon; IB: Iberian; KR: Krškopolje; LI: Lithuanian indigenous wattle; MA: Swallow-Bellied Mangalitsa; MB: Majorcan Black; MR: Mora Romagnola; MV: Moravka; NS: Nero Siciliano; OW: Old type Lithuanian White; SA: Sarda; SW: Schwäbisch-Hällisches Schwein;TU: Turopolje; WB: Wild Boar.

Besides, breed specific signatures were identified as those in the 99.9^th^ percentile of the empirical distributions specifically detected in one breed but not in the remaining ones (Supplementary Tables 48–69). A total of 115 breed specific regions were detected in the 21 populations, with a mean of 5 ± 3 regions detected per breed. The number of specific signatures detected in each breed was quite variable. Several breeds showed a large number of specific regions, such as Turopolje (14 regions) or Mora Romagnola (12 regions), while the numbers were smaller in other ones, such as Iberian or Sarda with just one region detected. Chromosomes 1 and 13 were the ones harboring the highest’s numbers of breed specific regions (15 and 17, respectively). The higher differentiation observed on Turopolje and Mora Romagnola may suggest a higher genetic drift in those breeds due to their limited effective population size^50^. The identified regions contain interesting candidate genes involved in functions and pathways related to productive and behavioral traits. Although a detailed discussion of the genes identified in each of the quoted regions is beyond the scope of this work, some findings can be highlighted.

Several different olfactory receptor genes were detected within the potentially selected regions in Apulo Calabrese (SSC9), Gascon (SSC13), Iberian (SSC15), Lithuanian Indigenous wattle (SSC4), Swallow-Bellied Mangalitsa (SSC2) and Mora Romagnola (SSC1). This is in agreement with the large repertoire of functional olfactory receptor genes in pigs, the fast evolution of these genes^33,51^ and their relevant role in smell and food finding, especially in these extensively-reared breeds. The putative selection of different subfamilies in each breed could match with the specialization in the detection of specific odors, characteristic of each breeds’ environment, as there is a wide functional diversity among olfactory receptors subfamilies. In fact, different gene clusters have been potentially associated with the recognition of specific odorants^42^. Moreover, the relevance of these genes exceeds their role in olfaction as they are also implicated in reproductive and behavioral traits, which may influence fitness.

Also, candidate genes involved in relevant metabolic functions associated with the adipogenic phenotype of our breeds are identified within the putative selected regions. For instance, in the Basque breed, the genes *ACOX1* and *CPT1A*, both involved in fatty acid metabolism are identified. *NFKBIA* and *PPARGC1B* which have both been associated with backfat thickness in pigs^52,53^ are detected in Krškopolje and Cinta Senese breeds, respectively. *DECR1*, a positional candidate gene for the first fatness QTL detected in pigs on SSC4^54,55^, is observed in a potentially selected region in Gascon breed. *DLK1*, a gene with a fundamental role in muscle growth and fat deposition^56^, is detected in Majorcan Black. *LPIN1*, which has been associated with obese pig phenotypes^57^, is detected in Turopolje breed. Taste receptor genes play a fundamental role in survival through the identification of dietary nutrients or potentially toxic substances, being linked to eating behavior and adaptation to specific geographical locations and diets^58^ and potentially related to growth and fat deposition^10,59^. Among this gene family, the *TAS2R16* gene was detected in a selection signature in the Turopolje breed. Genes involved in the endocrine regulation of growth and insulin signalling, are also observed: *IRS1* is detected in Casertana; *GAL* and *GALR2* in Basque, and members of the insulin like growth factor binding protein gene family are detected in Black Slavonian. These genes code for signalling molecules that integrate and coordinate numerous biologically key extracellular signals within the cell. Some of them are intermediate of the insulin signalling, with a key role in growth, fatness and energy homeostasis^60,61^.

Different and abundant genes involved in proteolysis were also found, such as *HEDTC2* and *IDE* in Alentejana; *USP54* in Casertana; *CAPN10*, *RNPEPL1* in Iberian; *PSMA6* in Krškopolje, *CTSV* in Turopolje, or *UBE4B*, *RNF150* and *UBE2E1* detected in 5 or more breeds simultaneously (Table 3). Increased protein turnover has been proposed as a potential determinant for the limited muscle growth usually observed in local pigs^2,62,63^. A signal of diversifying selection was reported by Wilkinson *et al*.^64^ and Ai *et al*.^65^ in European pig breeds, close to the *EDNRB* gene, which is implicated in coat color pattern in mammals. This same region has been detected in the present work in Apulo Calabrese breed. Nevertheless, signals of selection were not detected near the two main known coat color genes, *KIT* and *MC1R*, for which allelic variation is associated with many of the coat color variants in pigs^66,67^. This could be due to incomplete coverage or informativity of the SNP chip in these particular regions.

Regarding Wild Boar-specific selection signatures, only three regions were detected. Two of them are located in SSC7, an autosome that has been repeatedly shown to be associated with domestication and behavior-related traits in QTL and GWAS studies^68^. Interestingly, the *TECTB* gene is potentially included in a selected genome region in Wild Boar. This gene is expressed in the inner ear and has a main role in hearing, which may be associated with survival in wild environmental conditions. One of the regions detected in SSC7 (33.55–33.59 cM) is located very close to *PPARD* gene (31.22–31.29 cM), related to ear morphology, fat deposition and growth, and detected previously as being located in a differentiated genomic region between European breeds and Wild Boar^64^.

In previous works analyzing different commercial and traditional pig populations, a number of regions showing between-breed signatures of selection has been identified^16,64^. In these studies, as well as in the present one, genes mapped to these regions can be considered as candidates under selection in pig breeds. Some common biological functions have been detected in different works, such as olfaction, growth or muscle development^69,70^. Nevertheless, when comparing different works, variable regions and genes have been observed probably due to differences in the breeds analyzed, statistical methods, SNP density or sample size. Especially, domestic pigs under different evolution and production conditions show different selection signatures and in our case all tested populations are locally produced breeds, which have not been selected for lean meat content, muscularity or enhanced reproduction. Thus, in a differentiation analysis among those breeds, expected signatures may be weaker than those observed in commercial and highly selected genotypes, or more related to domestication and breed standards establishment than actual artificial selection processes.

## Conclusions

The obtained results were useful for the characterization of the genomic diversity of autochthonous European pig breeds. Results highlighted the genetic closeness among several of these domesticated breeds, and with their wild ancestor, as well as clear differentiation of some other ones and confirm the need of conservation programs to protect their genetic pools. Linkage disequilibrium patterns and extent have been determined at the genome level for a wide repertoire of European traditional breeds, showing potential effects of admixture and inbreeding. Putative signals of selection were detected for regions containing genes involved in growth, muscle development, reproduction, metabolism, behavior and sensory perception. Our findings improve the knowledge about the genome biology of European local pig breeds, and provide candidate genes for relevant traits, as well as useful information for future conservation, association or selection approaches.

## Methods

### Animals and sampling

Experts of each country, including personnel from breeding organizations and herd books, selected the animals to be included in the analyses in order to get a representative sampling of each breed. Selection of individuals for sampling was performed by avoiding highly related animals (no full- or half-sibs), balancing between sexes and prioritizing adult individuals or at least animals with adult morphology. Blood samples were obtained from 39 to 54 individuals from each one of the 20 local pig breeds included in the study: Black Slavonian and Turopolje (Croatia), Basque and Gascon (France), Schwäbisch-Hällisches Schwein (Germany), Apulo-Calabrese, Casertana, Cinta Senese, Mora Romagnola, Nero Siciliano and Sarda (Italy), Lithuanian indigenous wattle and Lithuanian White old type (Lithuania), Alentejana and Bísara (Portugal), Moravka and Swallow-Bellied Mangalitsa (Serbia), Krškopolje pig (Slovenia) and Iberian and Majorcan Black (Spain). Besides, seven European wild boars were employed as outgroup. Specialized professionals from each institution that provided animal material obtained blood samples following standard routine monitoring procedures and guidelines, at farm or at slaughter. No procedures with animals were performed that would demand ethical protocols according to Directive 2010/63/EU (2010) and blood samples were obtained as a general breeding procedure or previously collected DNA only reused here. A total of 992 DNA samples were genotyped.

The genomic DNA was extracted from leukocytes present in 8–15 mL of peripheral blood, collected in Vacutainer tubes containing 10% 0.5 M EDTA (ethylenediaminetetraacetic acid, disodium dihydrate salt) at pH 8.0. The extraction was performed using either a standardized phenol-chloroform, high-salt method or a commercial kit^71^.

### Genotyping

Samples were genotyped with the GeneSeek ® GGP Porcine HD Genomic Profiler v1 (Illumina Inc, USA), which includes 68,516 SNPs evenly distributed with a median of 25 kb gap spacing and sharing 42,372 markers with Illumina porcine SNP60 chip. The average genotyping call rate was 0.94.

Genotype quality control (QC) and data filtering were performed using PLINK^72^. SNPs with MAF lower than 0.01 or more than 10% missing genotypes were excluded from the analyses in a preliminary filter to inspect the distribution of MAF across the genotyped SNPs. In addition, an individual call rate threshold was set to 95% and ten samples (two Bísara, two Casertana, one Cinta Senese, one Moravka two Nero Siciliano and two Schwäbisch-Hällisches) were removed for further analyses.

### Data analyses

A total of 51,219 SNPs mapped on the 18 autosomes on Sus Scrofa 11.1 were used to compute, for the 20 studied breeds, the following indicators of genetic diversity: observed (H_O_) and expected heterozygosity (H_E_), inbreeding coefficient of an individual (I) relative to the subpopulation (S) (F_IS_), fixation index (F_ST_) and inbreeding coefficient of an individual (I) relative to the total (T) population (F_IT_), heterozygosity index based on observed heterozygosity in individuals within breeds (H_I_) based on expected heterozygosity in subpopulation (H_S_) and based on expected heterozygosity for overall breeds (H_T_). Calculations were made with VCFtools software^73^. Nei’s genetic distances^12^ were calculated in R^74^ environment. Pairwise distances were used to build a NJ tree with the *nj* function belonging to the *ape*^75^ library in R. In addition, population structure was inspected trough PCA analyses performed with DISSECT software tool^76^.

### Linkage disequilibrium (LD) analyses

Markers with MAF lower than 0.05, with more than a 10% of missing values, significantly deviating from Hardy-Weinberg equilibrium (P < 8.27 × 10^−7^) and unmapped or mapped in sexual chromosomes were excluded from LD analysis. This QC filtering was carried out independently for each breed and the number of SNP used is showed in Supplementary Table 3. The LD coefficient r^2^ was estimated for all marker pairs less than 50 Mb for each population and autosome independently using PLINK. To plot LD *vs* physical distance between markers, following Saura *et al*.^4^, we divided SNP pairs into three distance classes, (a) 0 to 2 Mb, (b) 2 to 5 Mb and (c) 5 to 50 Mb. Distance bins of 0.05, 0.20 and 5.0 Mb were used for classes (a), (b) and (c), respectively, and average r^2^ values for each bin were plotted against physical distance. Sample sizes were similar for all the breeds except for Wild Boar. As sample size can have an influence on LD estimation, a correction for sampling size was used to estimate r^2^ in Wild Boar as follows: (r^2^ − 1/N) (1 − 1/N)^77^.

### Effective population size across generations

Estimates of effective population size (N_e_) for each population were computed using the relationship between LD and N_e_ according to the following equation^39,78^:

$${r}^{2}={(\propto -4{{\bf{N}}}_{{\bf{e}}}{\bf{c}})}^{-{\rm{1}}}+1/{\rm{N}}$$

where **c** is the distance between SNPs (Morgans), **N**_**e**_ is the sample size and absence of mutation was assumed ($\propto =1$) and N is equal to the number of diploid individuals sampled in the analyses. r^2^ estimates were computed between all pairs within 50 Mb windows within chromosome. Physical distances were converted to genetic distances in Morgans taking into account the specific recombination rate estimated for each chromosome by Muñoz *et al*.^79^. To estimate population size per generation, r^2^ between SNP pairs at a determined specific genetic distance corresponding to t = 1/2c^80^, where **t** is the generation, was considered. Finally, N_e_ at each generation was estimated through a non-linear least square approach based on the equation mentioned above. N_e_ estimates were calculated back for a period of 50 generations based on the same set of genotypes used in the linkage disequilibrium analyses (Supplementary Table 3).

### F_ST_ analyses

Hardy-Weinberg equilibrium was inspected individually in all breeds. SNPs significantly deviating from Hardy-Weinberg equilibrium (P < 8.27 × 10^−7^) in at least one of the studied breeds were removed. A total of 828 SNPs was extracted from the subset of 51,219 SNPs used to compute genetic diversity parameters. Pairwise Wright’s F_ST_^81^ were estimated as a measure of genetic differentiation according to the method described in Wilkinson *et al*.^64^. For each breed, a total of 21 breed-pairwise comparisons at each SNP were obtained and they were averaged to get overall F_ST_ for each SNP per breed. F_ST_ values were averaged in sliding windows of 13 SNPs centred at the 7^th^ SNP which determined the genomic location of the window. Regions in the 99^th^ percentile of the empirical distributions of windows per breed were defined as candidate regions to genetic differentiation. Genes were annotated with Biomart tool^82^.

## Supplementary information

Supplementary material 1

Supplementary material 2

Supplementary material 3

Supplementary material 4

## Data Availability

The authors confirm that the data supporting the findings of this study are available within the article and its supplementary materials. The raw genetic datasets generated during the current study are available from the corresponding author on reasonable request.
