# Supplementary material for: Genomic diversity, linkage disequilibrium and selection signatures in European local pig breeds assessed with a high density SNP chip
Source: Sci Rep. 2019 Sep 19;9:13546. doi: 10.1038/s41598-019-49830-6 (PMC6753209; doi:10.1038/s41598-019-49830-6)
Supplement: Supplementary file 1 — Supplementary material 1 [file 41598_2019_49830_MOESM1_ESM.pdf]

Genomic diversity, linkage disequilibrium and selection signatures in European local pig breeds assessed with a high density SNP chip

Muñoz M, Bozzi R, García-Casco J, Núñez Y, Ribani A, Franci O, García F, Škrlep M, Schiavo G, Bovo S, Utzeri VJ, Charneca R, Martins JM, Quintanilla R, Tibau J, Margeta V, Djurkin-Kušec I, Mercat MJ, Riquet J, Estellé J, Zimmer C, Razmaite V, Araujo JP, Radović Č, Savić R, Karolyi D, Gallo M, Čandek-Potokar M, Fernández AI, Fontanesi L, Óvilo C

Supplementary Tables Legends

Supplementary Table 1. Minimum allele frequency (MAF) ranges computed for each SNP by breed. (PDF)

Supplementary Table 2. Genetic distances among the 21 breeds estimated using Nei's formula (1972). (PDF)

Supplementary Table 3. SNPs used in LD decay analyses by population. (PDF)

Supplementary Table 4. Average r^2 values and standard deviation at a given distance intervals for each breed. (XLS)

Supplementary Table 5. Recombination rate averaged by chromosome and r^2 averaged at distances of 0.05 Mb per population and chromosome. (XLS)

Supplementary Table 6. Effective population sizes and their standard errors estimated along 50 generations corresponding to Alentejana breed. (XLS)

Supplementary Table 7. Effective population sizes and their standard errors estimated along 50 generations corresponding to Apulo Calabrese breed. (XLS)

Supplementary Table 8. Effective population sizes and their standard errors estimated along 50 generations corresponding to Basque breed. (XLS)

Supplementary Table 9. Effective population sizes and their standard errors estimated along 50 generations corresponding to Bísara breed. (XLS)

Supplementary Table 10. Effective population sizes and their standard errors estimated along 50 generations corresponding to Black Slavonian breed. (XLS)

Supplementary Table 11. Effective population sizes and their standard errors estimated along 50 generations corresponding to Casertana breed. (XLS)

Supplementary Table 12. Effective population sizes and their standard errors estimated along 50 generations corresponding to Cinta Senese breed. (XLS)

Supplementary Table 13. Effective population sizes and their standard errors estimated along 50 generations corresponding to Gascon breed. (XLS)

Supplementary Table 14. Effective population sizes and their standard errors estimated along 50 generations corresponding to Iberian breed. (XLS)

Supplementary Table 15. Effective population sizes and their standard errors estimated along 50 generations corresponding to Krškopolje breed. (XLS)

Supplementary Table 16. Effective population sizes and their standard errors estimated along 50 generations corresponding to Lithuanian indigenous wattle breed. (XLS)

Supplementary Table 17. Effective population sizes and their standard errors estimated along 50 generations corresponding to Majorcan Black breed. (XLS)

Supplementary Table 18. Effective population sizes and their standard errors estimated along 50 generations corresponding to Mangalitsa breed. (XLS)

Supplementary Table 19. Effective population sizes and their standard errors estimated along 50 generations corresponding to Mora Romagnola breed. (XLS)

Supplementary Table 20. Effective population sizes and their standard errors estimated along 50 generations corresponding to Moravka breed. (XLS)

Supplementary Table 21. Effective population sizes and their standard errors estimated along 50 generations corresponding to Nero Siciliano breed. (XLS)

Supplementary Table 22. Effective population sizes and their standard errors estimated along 50 generations corresponding to Old Type Lithuanian White breed. (XLS)

Supplementary Table 23. Effective population sizes and their standard errors estimated along 50 generations corresponding to Sarda breed. (XLS)

Supplementary Table 24. Effective population sizes and their standard errors estimated along 50 generations corresponding to Schwäbisch-Hällisches Schwein breed. (XLS)

Supplementary Table 25. Effective population sizes and their standard errors estimated along 50 generations corresponding to Turopolje breed. (XLS)

Supplementary Table 26. Effective population sizes and their standard errors estimated along 50 generations corresponding to Wild boar. (XLS)

Supplementary Table 27. Outlier windows found in the 99th percentile of F_{ST} values identified in Alentejana breed. (XLS)

Supplementary Table 28. Outlier windows found in the 99th percentile of F_{ST} values identified in Apulo Calabrese breed. (XLS)

Supplementary Table 29. Outlier windows found in the 99th percentile of F_{ST} values identified in Basque breed. (XLS)

Supplementary Table 30. Outlier windows found in the 99th percentile of F_{ST} values identified in Bísara breed. (XLS)

Supplementary Table 31. Outlier windows found in the 99th percentile of F_{ST} values identified in Black Slavonian breed. (XLS)

Supplementary Table 32. Outlier windows found in the 99th percentile of F_{ST} values identified in Casertana breed. (XLS)

Supplementary Table 33. Outlier windows found in the 99th percentile of F_{ST} values identified in Cinta Senese breed. (XLS)

Supplementary Table 34. Outlier windows found in the 99th percentile of F_{ST} values identified in Gascon breed. (XLS)

Supplementary Table 35. Outlier windows found in the 99th percentile of F_{ST} values identified in Iberian breed. (XLS)

Supplementary Table 36. Outlier windows found in the 99th percentile of F_{ST} values identified in Krškopolje breed. (XLS)

Supplementary Table 37. Outlier windows found in the 99th percentile of F_{ST} values identified in Lithuanian indigenous wattle breed. (XLS)

Supplementary Table 38. Outlier windows found in the 99th percentile of F_{ST} values identified in Majorcan Black breed. (XLS)

Supplementary Table 39. Outlier windows found in the 99th percentile of F_{ST} values identified in Mangalitsa breed. (XLS)

Supplementary Table 40. Outlier windows found in the 99th percentile of F_{ST} values identified in Mora Romagnola breed. (XLS)

Supplementary Table 41. Outlier windows found in the 99th percentile of F_{ST} values identified in Moravka breed. (XLS)

Supplementary Table 42. Outlier windows found in the 99th percentile of F_{ST} values identified in Nero Siciliano breed. (XLS)

Supplementary Table 43. Outlier windows found in the 99th percentile of F_{ST} values identified in Sarda breed. (XLS)

Supplementary Table 44. Outlier windows found in the 99th percentile of F_{ST} values identified in Schwäbisch-Hällisches Schwein breed. (XLS)

Supplementary Table 45. Outlier windows found in the 99th percentile of F_{ST} values identified in Old type Lithuanian White breed. (XLS)

Supplementary Table 46. Outlier windows found in the 99th percentile of F_{ST} values identified in Turopolje breed. (XLS)

Supplementary Table 47. Outlier windows found in the 99th percentile of F_{ST} values identified in Wild Boar. (XLS)

Supplementary Table 48. Genomic regions with outlier FST-windows specific of each breed (99.9th percentile) that are non-shared with the remaining ones. (XLS)

Supplementary Table 49. Total number of genomic regions with outlier FST-windows specific of Alentejana breed (99.9th percentile) that are non-shared with the remaining ones, and genes annotated within these regions in Sscrofa11.1. (XLS)

Supplementary Table 50. Total number of genomic regions with outlier FST-windows specific of Apulo Calabrese breed (99.9th percentile) that are non-shared with the remaining ones, and genes annotated within these regions in Sscrofa11.1. (XLS)

Supplementary Table 51. Total number of genomic regions with outlier FST-windows specific of Basque breed (99.9th percentile) that are non-shared with the remaining ones, and genes annotated within these regions in Sscrofa11.1. (XLS)

Supplementary Table 52. Total number of genomic regions with outlier FST-windows specific of Bísara breed (99.9th percentile) that are non-shared with the remaining ones, and genes annotated within these regions in Sscrofa11.1. (XLS)

Supplementary Table 53. Total number of genomic regions with outlier FST-windows specific of Black Slavonian breed (99.9th percentile) that are non-shared with the remaining ones, and genes annotated within these regions in Sscrofa11.1. (XLS)

Supplementary Table 54. Total number of genomic regions with outlier FST-windows specific of Casertana breed (99.9th percentile) that are non-shared with the remaining ones, and genes annotated within these regions in Sscrofa11.1. (XLS)

Supplementary Table 55. Total number of genomic regions with outlier FST-windows specific of Cinta Senese breed (99.9th percentile) that are non-shared with the remaining ones, and genes annotated within these regions in Sscrofa11.1. (XLS)

Supplementary Table 56. Total number of genomic regions with outlier FST-windows specific of Gascon breed (99.9th percentile) that are non-shared with the remaining ones, and genes annotated within these regions in Sscrofa11.1. (XLS)

Supplementary Table 57. Total number of genomic regions with outlier FST-windows specific of Iberian breed (99.9th percentile) that are non-shared with the remaining ones, and genes annotated within these regions in Sscrofa11.1. (XLS)

Supplementary Table 58. Total number of genomic regions with outlier FST-windows specific of Krškopolje breed (99.9th percentile) that are non-shared with the remaining ones, and genes annotated within these regions in Sscrofa11.1. (XLS)

Supplementary Table 59. Total number of genomic regions with outlier FST-windows specific of Lithuanian Indigenous Wattle breed (99.9th percentile) that are non-shared with the remaining ones, and genes annotated within these regions in Sscrofa11.1. (XLS)

Supplementary Table 60. Total number of genomic regions with outlier FST-windows specific of Mangalitsa breed (99.9th percentile) that are non-shared with the remaining ones, and genes annotated within these regions in Sscrofa11.1. (XLS)

Supplementary Table 61. Total number of genomic regions with outlier FST-windows specific of Majorcan Black breed (99.9th percentile) that are non-shared with the remaining ones, and genes annotated within these regions in Sscrofa11.1. (XLS)

Supplementary Table 62. Total number of genomic regions with outlier FST-windows specific of Mora Romagnola breed (99.9th percentile) that are non-shared with the remaining ones, and genes annotated within these regions in Sscrofa11.1. (XLS)

Supplementary Table 63. Total number of genomic regions with outlier FST-windows specific of Moravka breed (99.9th percentile) that are non-shared with the remaining ones, and genes annotated within these regions in Sscrofa11.1. (XLS)

Supplementary Table 64. Total number of genomic regions with outlier FST-windows specific of Nero Siciliano breed (99.9th percentile) that are non-shared with the remaining ones, and genes annotated within these regions in Sscrofa11.1. (XLS)

Supplementary Table 65. Total number of genomic regions with outlier FST-windows specific of Sarda breed (99.9th percentile) that are non-shared with the remaining ones, and genes annotated within these regions in Sscrofa11.1. (XLS)

Supplementary Table 66. Total number of genomic regions with outlier FST-windows specific of Old Type Lithuanian White (99.9th percentile) that are non-shared with the remaining ones, and genes annotated within these regions in Sscrofa11.1. (XLS)

Supplementary Table 67. Total number of genomic regions with outlier FST-windows specific of Schwäbisch-Hällisches breed (99.9th percentile) that are non-shared with the remaining ones, and genes annotated within these regions in Sscrofa11.1. (XLS)

Supplementary Table 68. Total number of genomic regions with outlier FST-windows specific of Turopolje breed (99.9th percentile) that are non-shared with the remaining ones, and genes annotated within these regions in Sscrofa11.1. (XLS)

Supplementary Table 69. Total number of genomic regions with outlier FST-windows specific of Wild Boar (99.9th percentile) that are non-shared with the remaining ones, and genes annotated within these regions in Sscrofa11.1. (XLS)

Supplementary Figures Legends

Supplementary Figure 1. Distribution of Heterozigosity Index based on observed (HI) and expected heterozigosities by populations (HS), total heterozygosity (HT), inbreeding coefficient of an individual (I) relative to the subpopulation (S) (FIS), fixation index (FST) and inbreeding coefficient of an individual (I) relative to the total (T) population (FIT). (PDF, page 2)

Supplementary Figure 2: Genome wide distribution of F_{ST} estimated in sliding windows in Alentejana breed. The continued red line delimits the 99th percentile. (PDF, page 3)

Supplementary Figure 3: Genome wide distribution of F_{ST} estimated in sliding windows in Apulo Calabrese breed. The continued red line delimits the 99th percentile. (PDF, page 4)

Supplementary Figure 4: Genome wide distribution of F_{ST} estimated in sliding windows in Basque breed. The continued red line delimits the 99th percentile. (PDF, page 5)

Supplementary Figure 5: Genome wide distribution of F_{ST} estimated in sliding windows in Bísara breed. The continued red line delimits the 99th percentile. (PDF, page 6)

Supplementary Figure 6: Genome wide distribution of F_{ST} estimated in sliding windows in Black Slavonian breed. The continued red line delimits the 99th percentile. (PDF, page 7)

Supplementary Figure 7: Genome wide distribution of F_{ST} estimated in sliding windows in Casertana breed. The continued red line delimits the 99th percentile. (PDF, page 8)

Supplementary Figure 8: Genome wide distribution of F_{ST} estimated in sliding windows in Cinta Senese breed. The continued red line delimits the 99th percentile. (PDF, page 9)

Supplementary Figure 9: Genome wide distribution of F_{ST} estimated in sliding windows in Gascon breed. The continued red line delimits the 99th percentile. (PDF, page 10)

Supplementary Figure 10: Genome wide distribution of F_{ST} estimated in sliding windows in Iberian breed. The continued red line delimits the 99th percentile. (PDF, page 11)

Supplementary Figure 11: Genome wide distribution of F_{ST} estimated in sliding windows in Krškopolje breed. The continued red line delimits the 99th percentile. (PDF, page 12)

Supplementary Figure 12: Genome wide distribution of F_{ST} estimated in sliding windows in Lithuanian indigenous wattle breed. The continued red line delimits the 99th percentile. (PDF, page 13)

Supplementary Figure 13: Genome wide distribution of F_{ST} estimated in sliding windows in Majorcan Black breed. The continued red line delimits the 99th percentile. (PDF, page 14)

Supplementary Figure 14: Genome wide distribution of F_{ST} estimated in sliding windows in Mangalitsa breed. The continued red line delimits the 99th percentile. (PDF, page 15)

Supplementary Figure 15: Genome wide distribution of F_{ST} estimated in sliding windows in Mora Romagnola breed. The continued red line delimits the 99th percentile. (PDF, page 16)

Supplementary Figure 16: Genome wide distribution of F_{ST} estimated in sliding windows in Moravka breed. The continued red line delimits the 99th percentile. (PDF, page 17)

Supplementary Figure 17: Genome wide distribution of F_{ST} estimated in sliding windows in Nero Siciliano breed. The continued red line delimits the 99th percentile. (PDF, page 18)

Supplementary Figure 18: Genome wide distribution of F_{ST} estimated in sliding windows in Old Type Lithuanian White breed. The continued red line delimits the 99th percentile. (PDF, page 19)

Supplementary Figure 19: Genome wide distribution of F_{ST} estimated in sliding windows in Sarda breed. The continued red line delimits the 99th percentile. (PDF, page 20)

Supplementary Figure 20: Genome wide distribution of F_{ST} estimated in sliding windows in A Schwaebisch−Haellisches Schwein breed. The continued red line delimits the 99th percentile. (PDF, page 21)

Supplementary Figure 21: Genome wide distribution of F_{ST} estimated in sliding windows in Turopolje breed. The continued red line delimits the 99th percentile. (PDF, page 22)

Supplementary Figure 22: Genome wide distribution of F_{ST} estimated in sliding windows in Wild Boar. The continued red line delimits the 99th percentile. (PDF, page 23)
