# Supplementary material for: Genomic diversity, linkage disequilibrium and selection signatures in European local pig breeds assessed with a high density SNP chip
Source: Sci Rep. 2019 Sep 19;9:13546. doi: 10.1038/s41598-019-49830-6 (PMC6753209; doi:10.1038/s41598-019-49830-6)

Genomic diversity, linkage disequilibrium and selection signatures in European local pig breeds assessed with a high density SNP chip

Muñoz M, Bozzi R, García-Casco J, Núñez Y, Ribani A, Franci O, García F, Škrlep M, Schiavo G, Bovo S, Utzeri VJ, Charneca R, Martins JM, Quintanilla R, Tibau J, Margeta V, Djurkin-Kušec I, Mercat MJ, Riquet J, Estellé J, Zimmer C, Razmaite V, Araujo JP, Radović Č, Savić R, Karolyi D, Gallo M, Čandek-Potokar M, Fernández AI, Fontanesi L, Óvilo C

Supplementary Figure 1. Distribution of Heterozigosity Index based on observed (HI) and expected heterozigosities by populations (HS), total heterozygosity (HT), inbreeding coefficient of an individual (I) relative to the subpopulation (S) (FIS), fixation index (FST) and inbreeding coefficient of an individual (I) relative to the total (T) population (FIT).

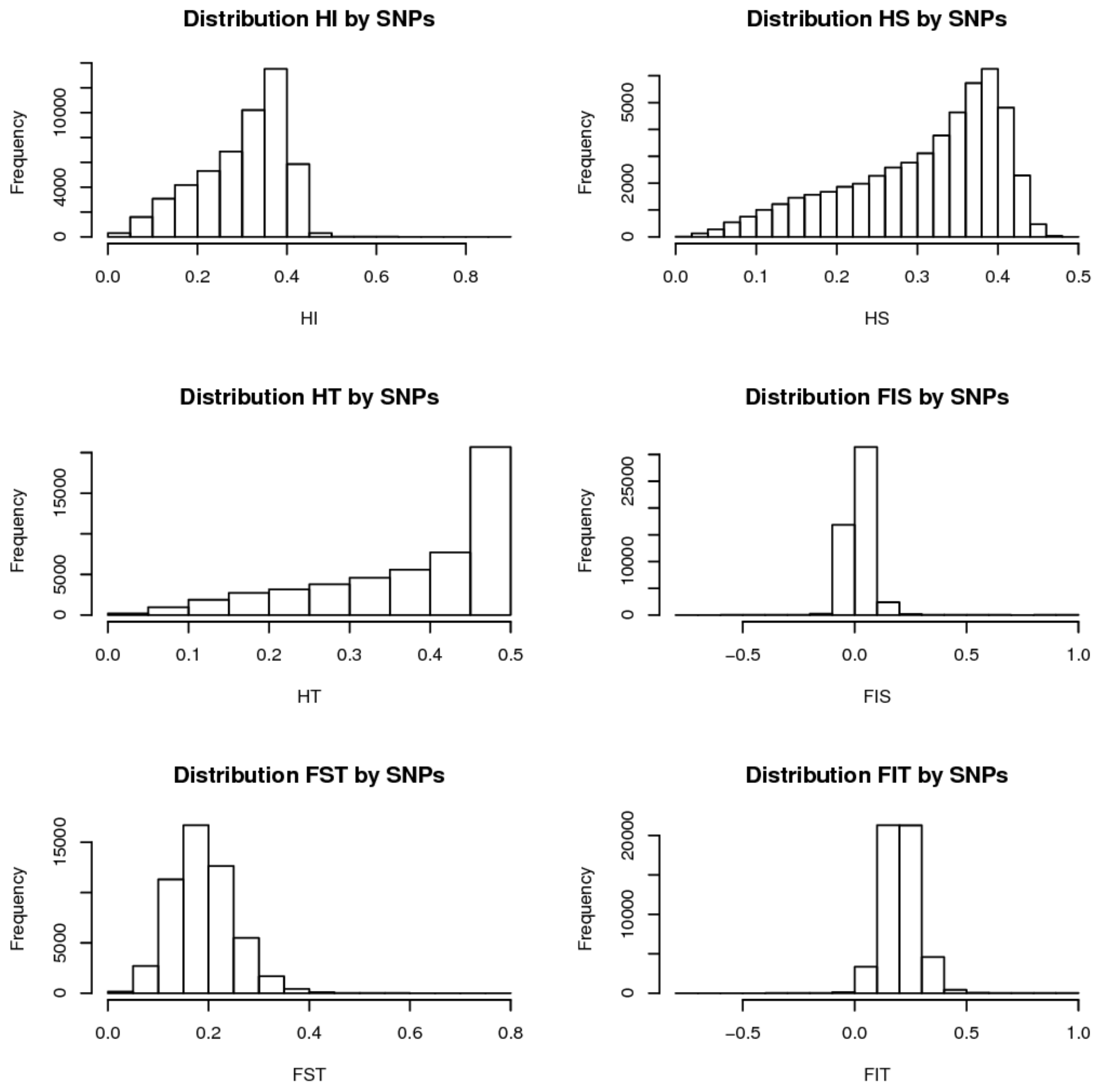

Supplementary Figure 2. Genome wide distribution of FST estimated in sliding windows in Alentejana breed.
The continued red line delimits the 99th percentile

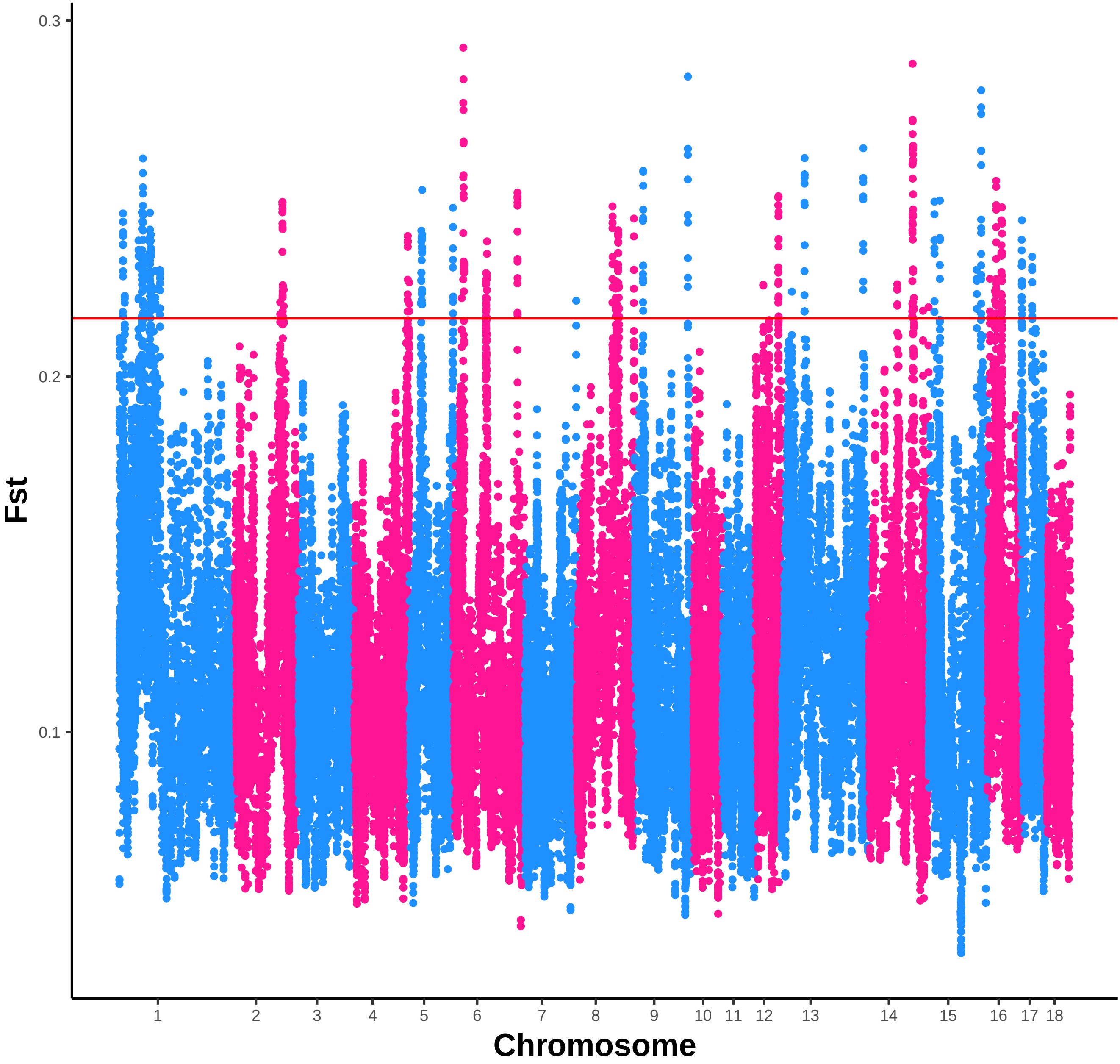

Supplementary Figure 3. Genome wide distribution of FST estimated in sliding windows in Apulo Calabrese breed. The continued red line delimits the 99th percentile

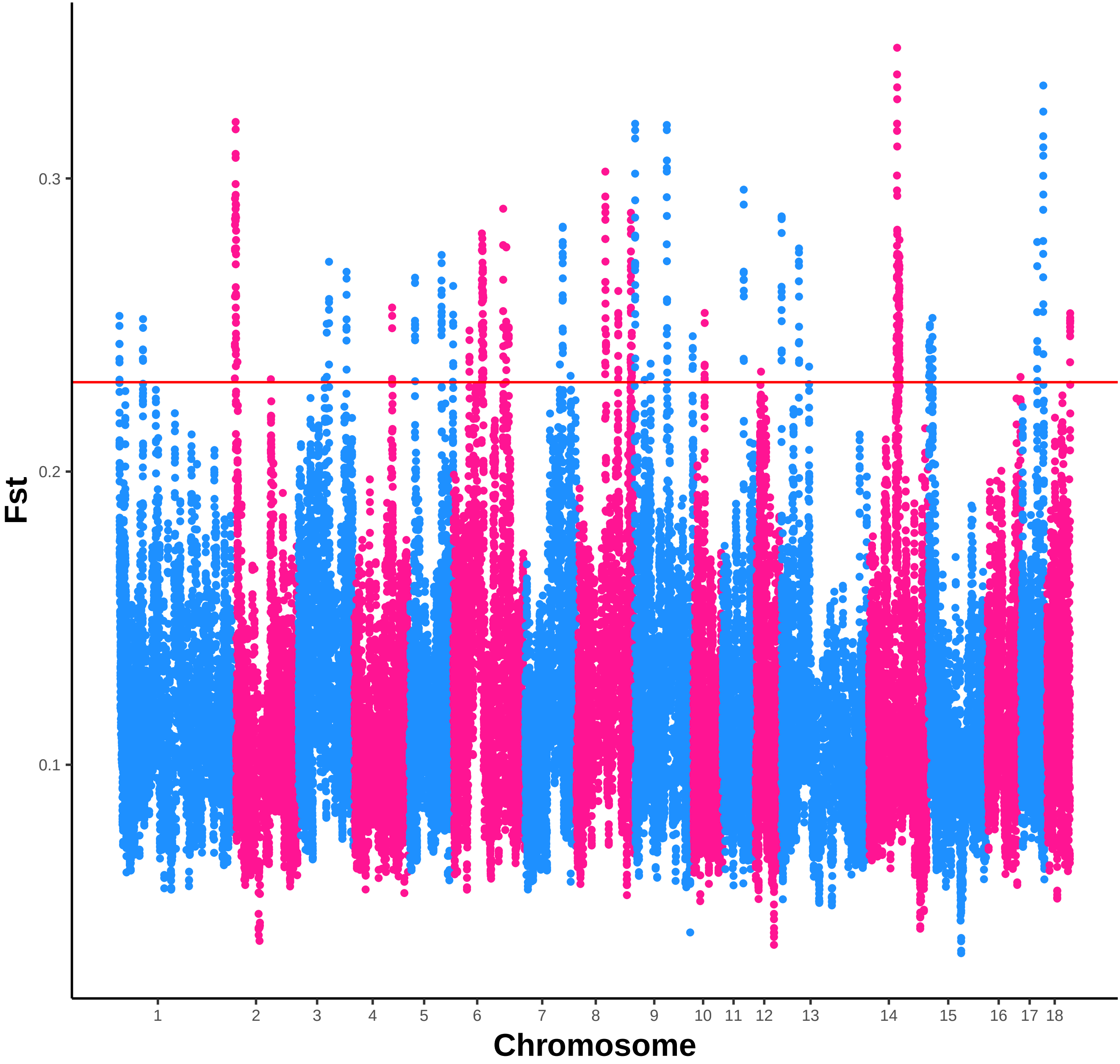

Supplementary Figure 4. Genome wide distribution of FST estimated in sliding windows in Basque breed. The continued red line delimits the 99th percentile

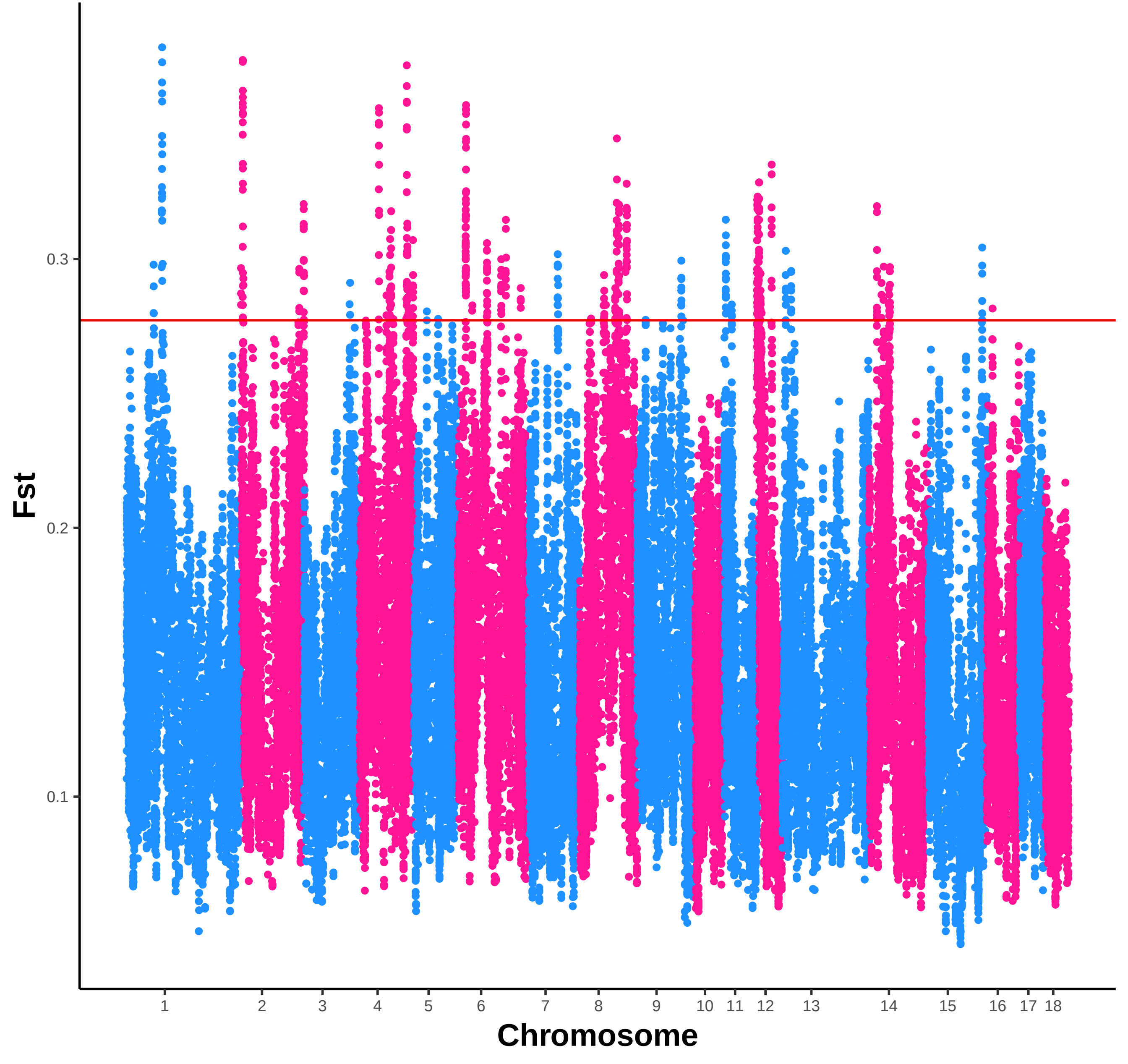

Supplementary Figure 5. Genome wide distribution of FST estimated in sliding windows in Bísara breed.
The continued red line delimits the 99th percentile

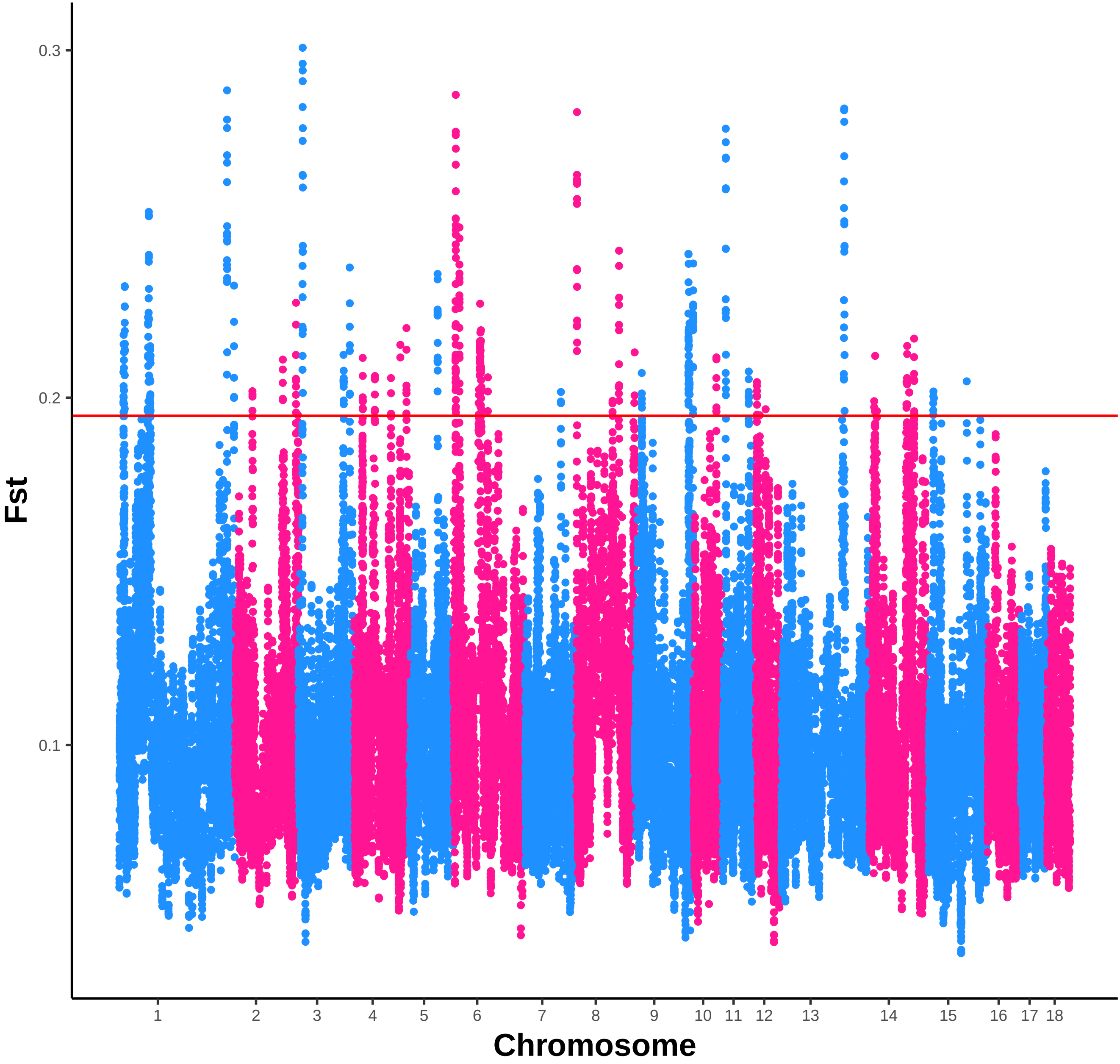

Supplementary Figure 6. Genome wide distribution of FST estimated in sliding windows in Black Slavonian breed. The continued red line delimits the 99th percentile

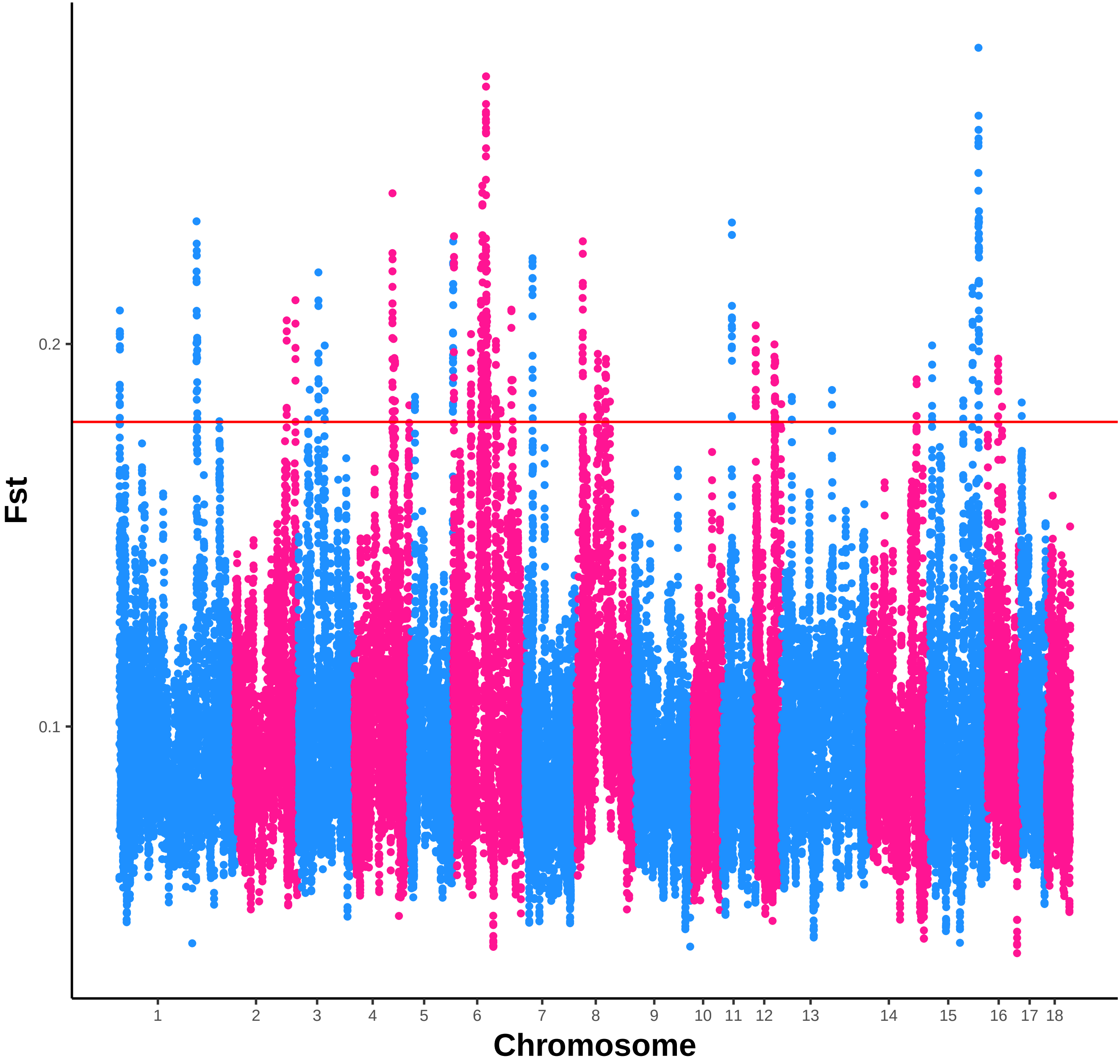

Supplementary Figure 7. Genome wide distribution of FST estimated in sliding windows in Casertana breed. The continued red line delimits the 99th percentile

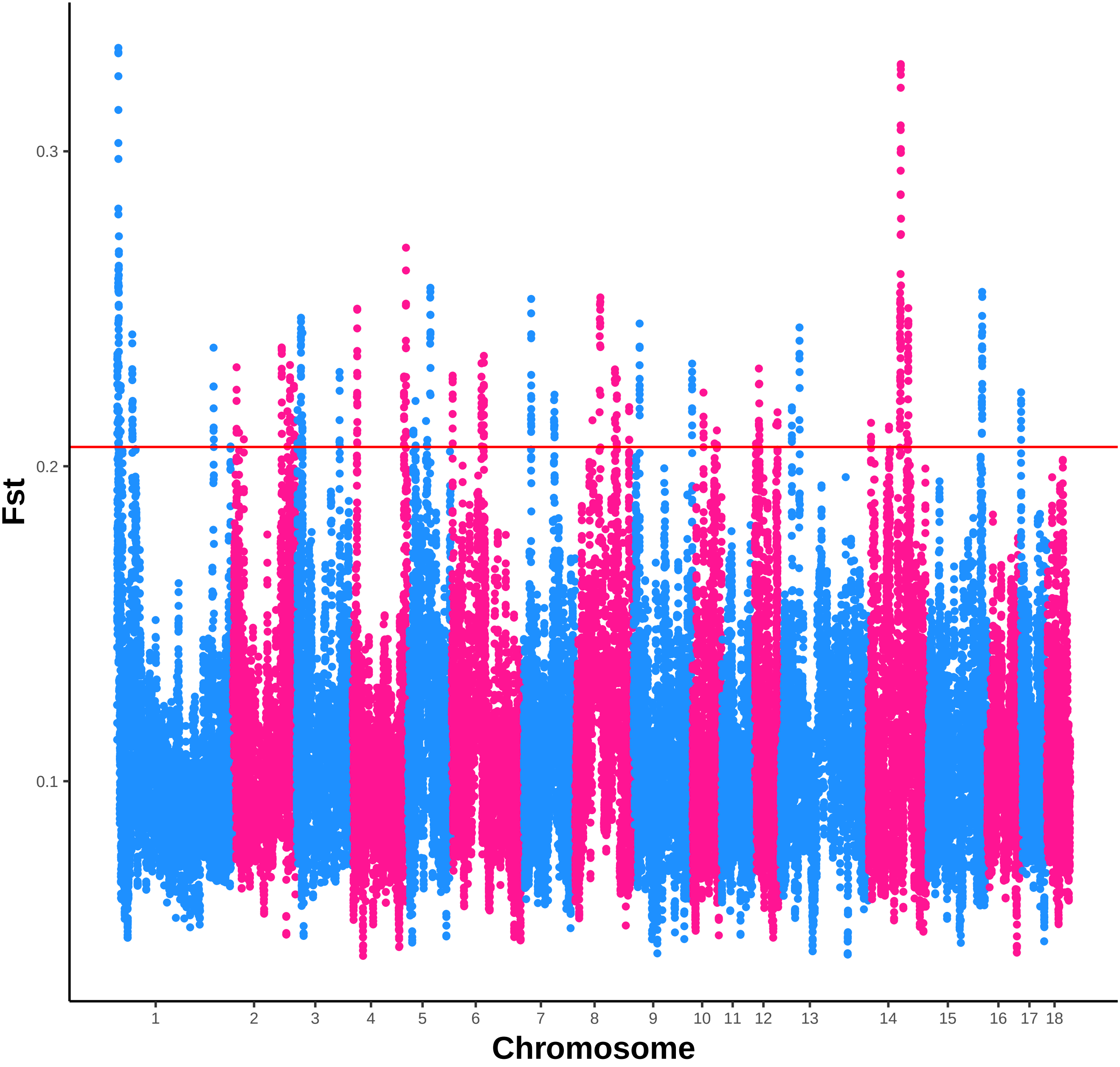

Supplementary Figure 8. Genome wide distribution of FST estimated in sliding windows in Cinta Senese breed.
The continued red line delimits the 99th percentile

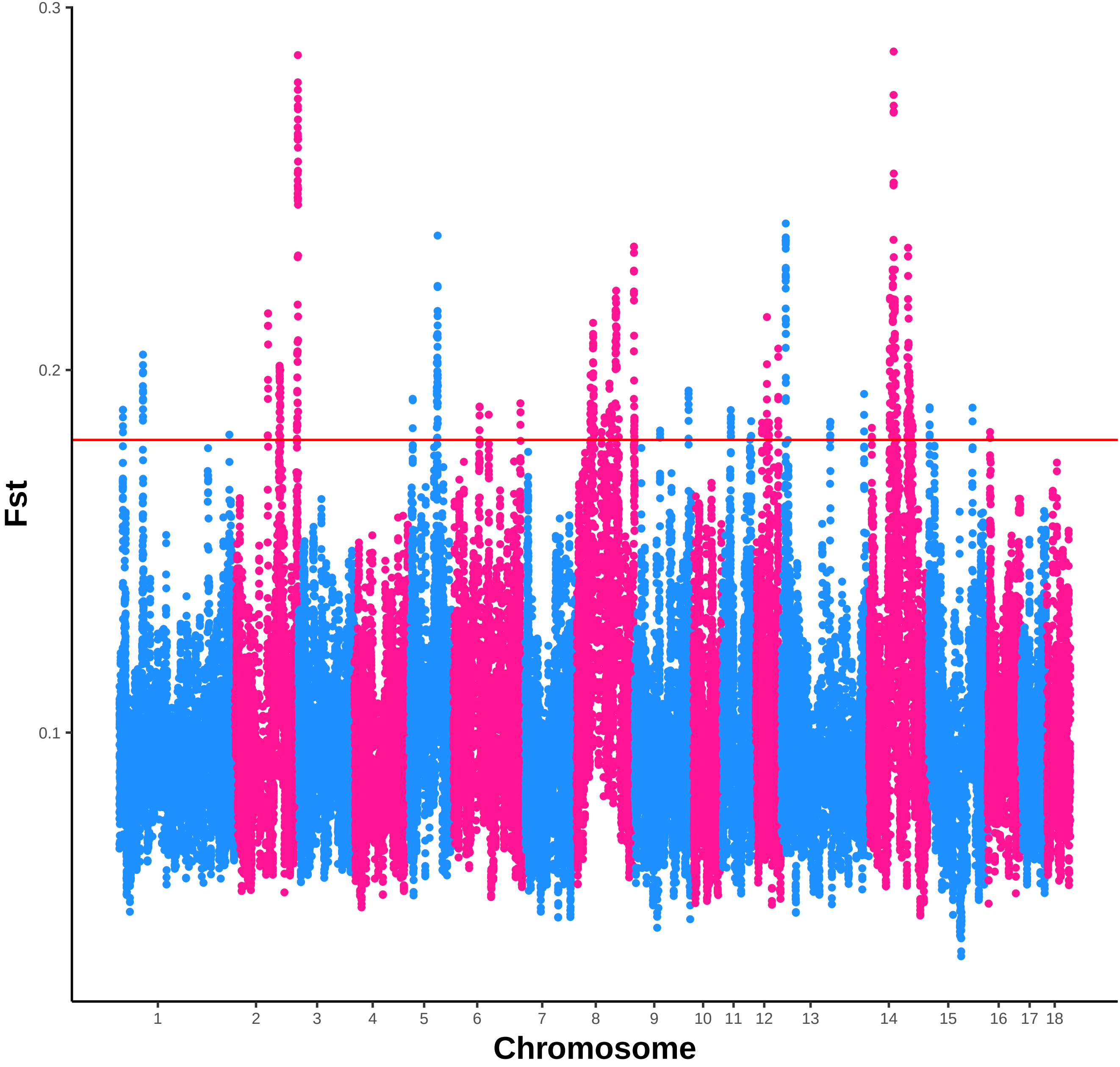

Supplementary Figure 9. Genome wide distribution of FST estimated in sliding windows in Gascon breed.
The continued red line delimits the 99th percentile

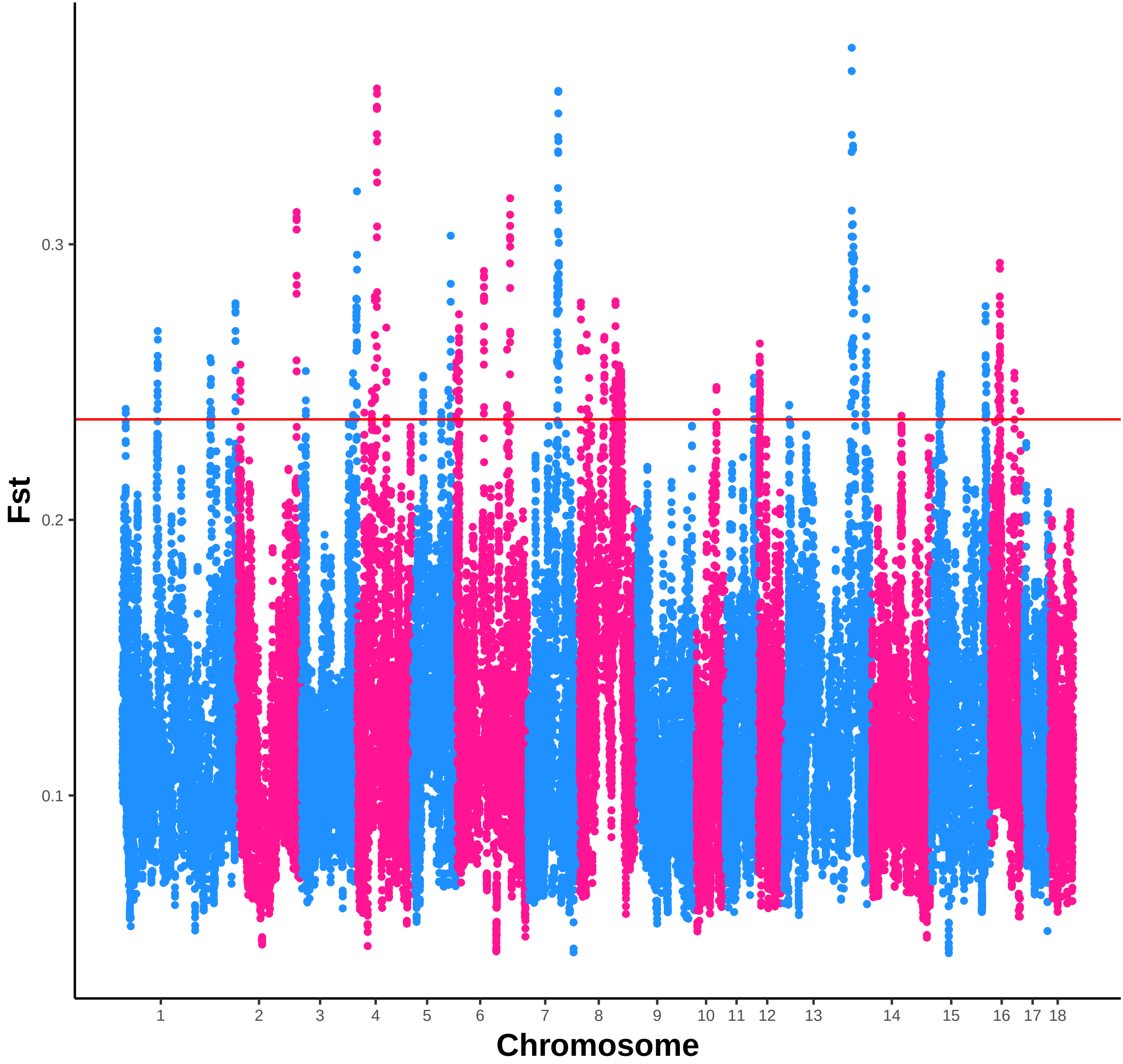

Supplementary Figure 10. Genome wide distribution of FST estimated in sliding windows in Iberian breed.
The continued red line delimits the 99th percentile

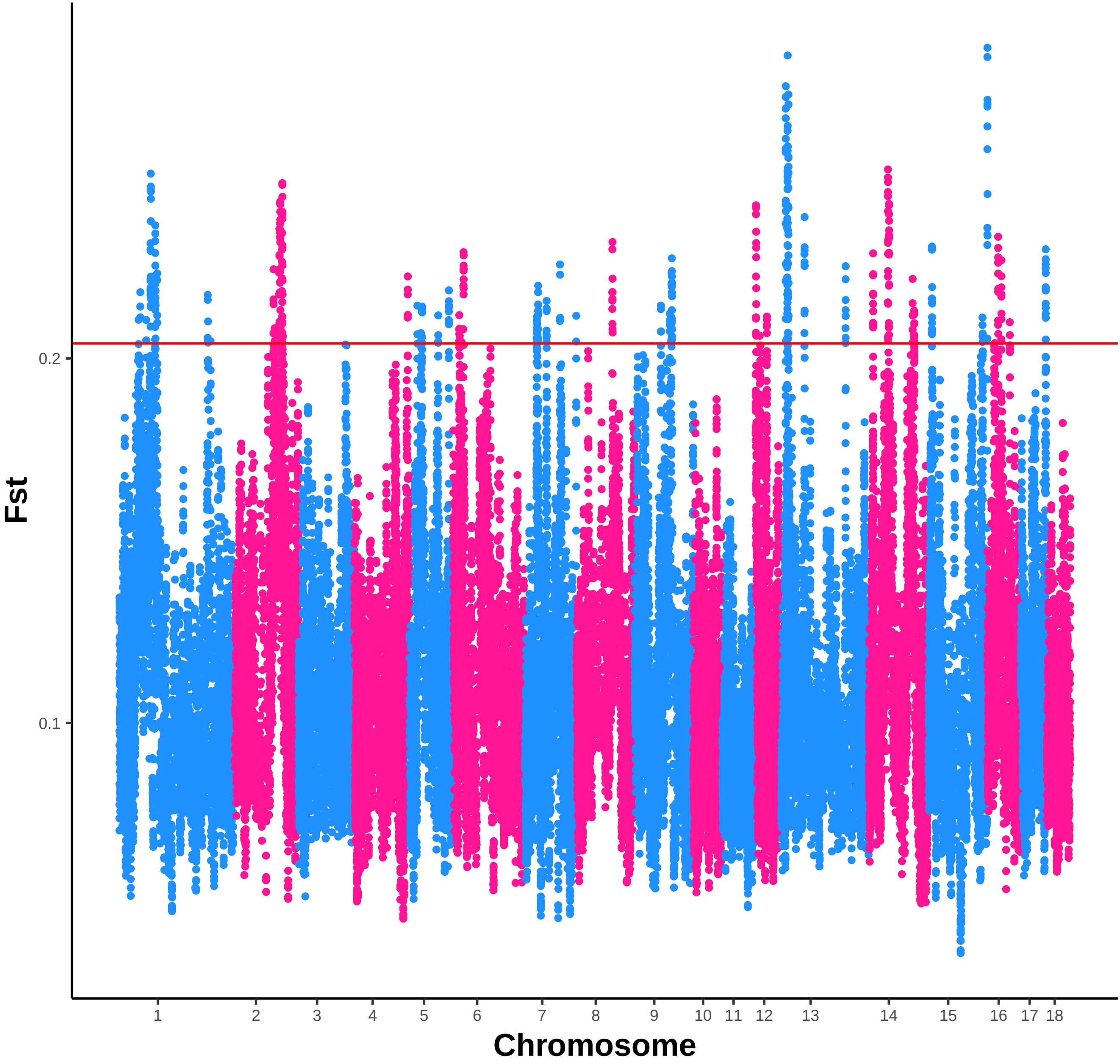

Supplementary Figure 11. Genome wide distribution of FST estimated in sliding windows in Krškopolje breed. The continued red line delimits the 99th percentile

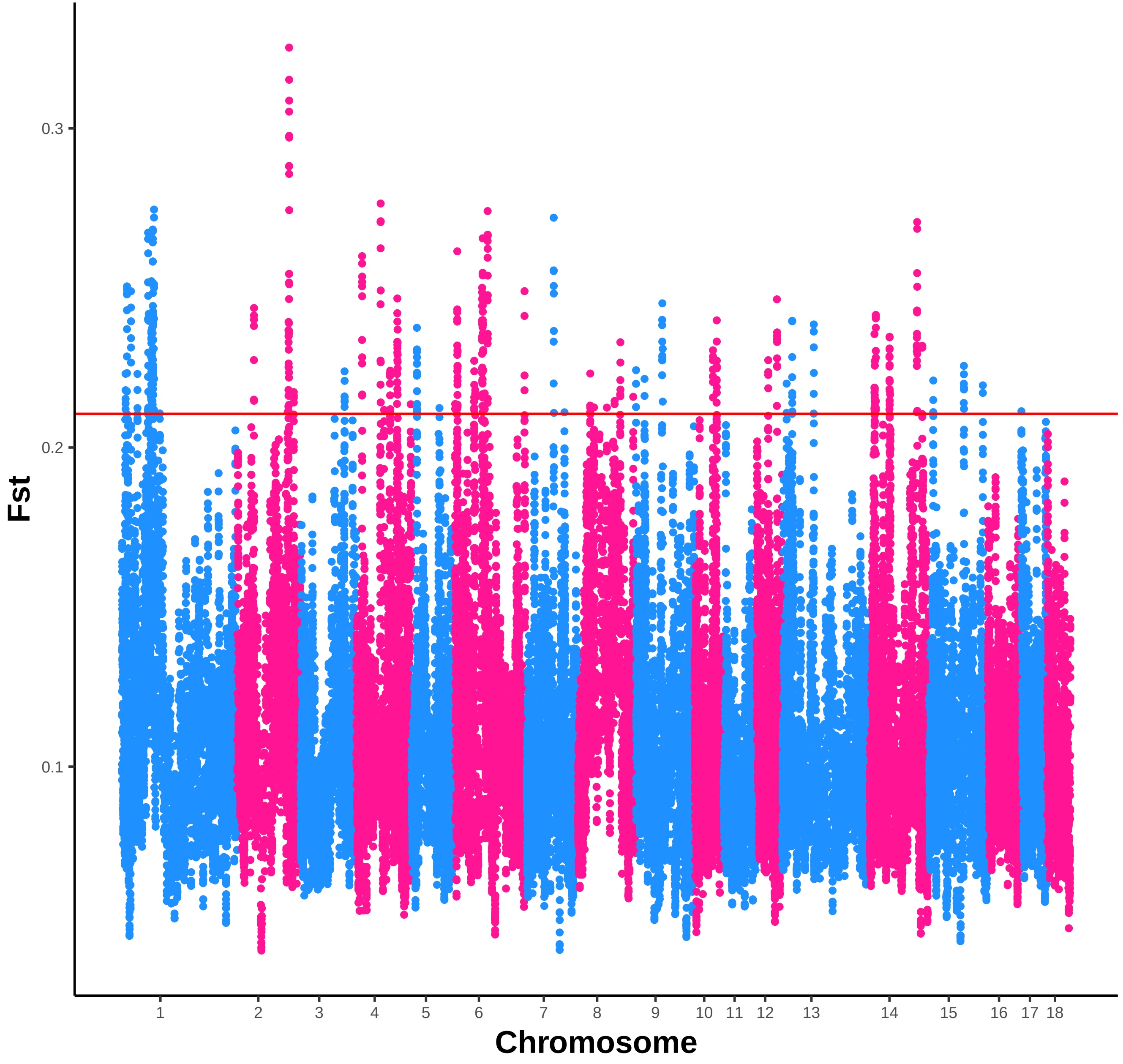

Supplementary Figure 12. Genome wide distribution of FST estimated in sliding windows in Lithuanian indigenous wattle breed. The continued red line delimits the 99th percentile

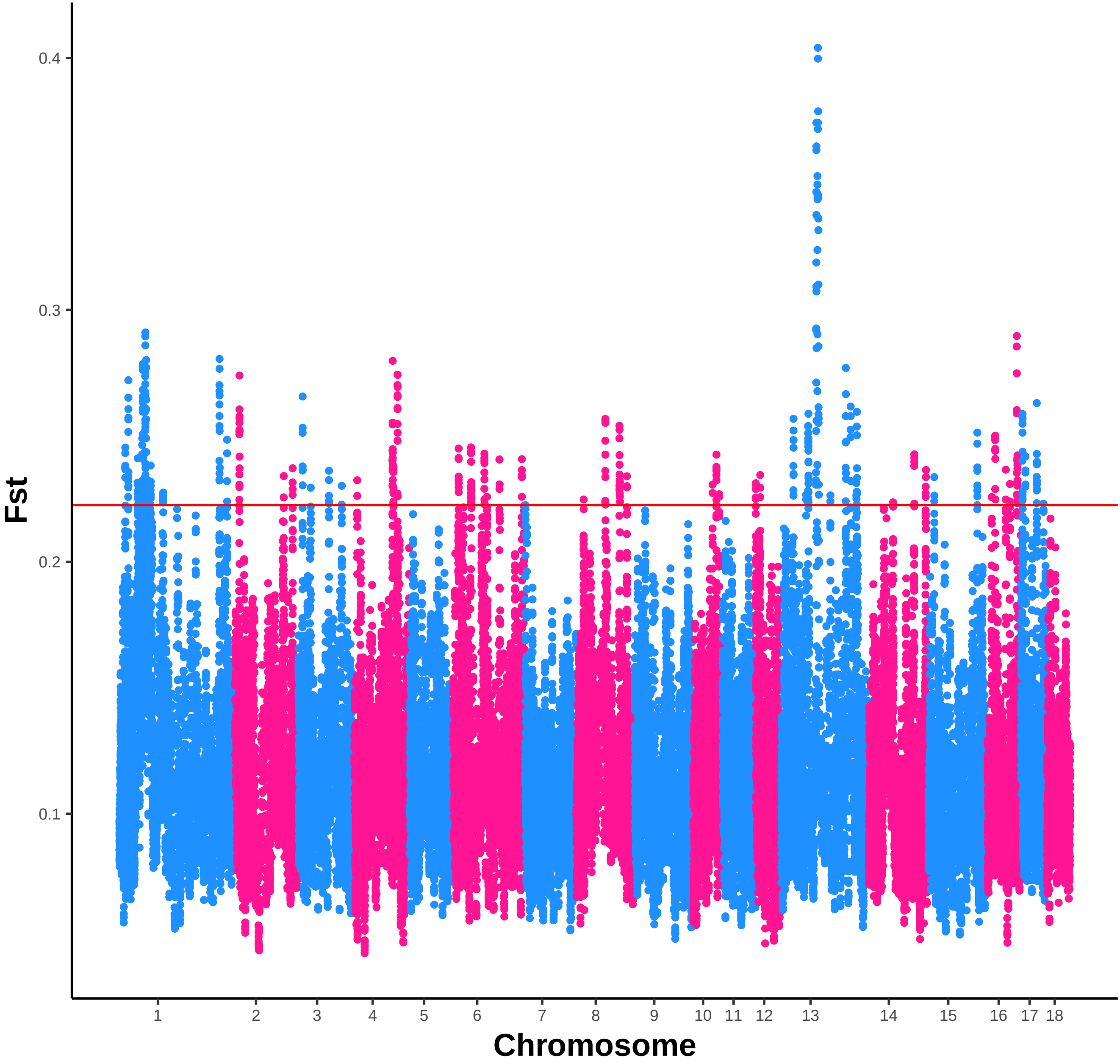

Supplementary Figure 13. Genome wide distribution of FST estimated in sliding windows in Majorcan Black breed. The continued red line delimits the 99th percentile

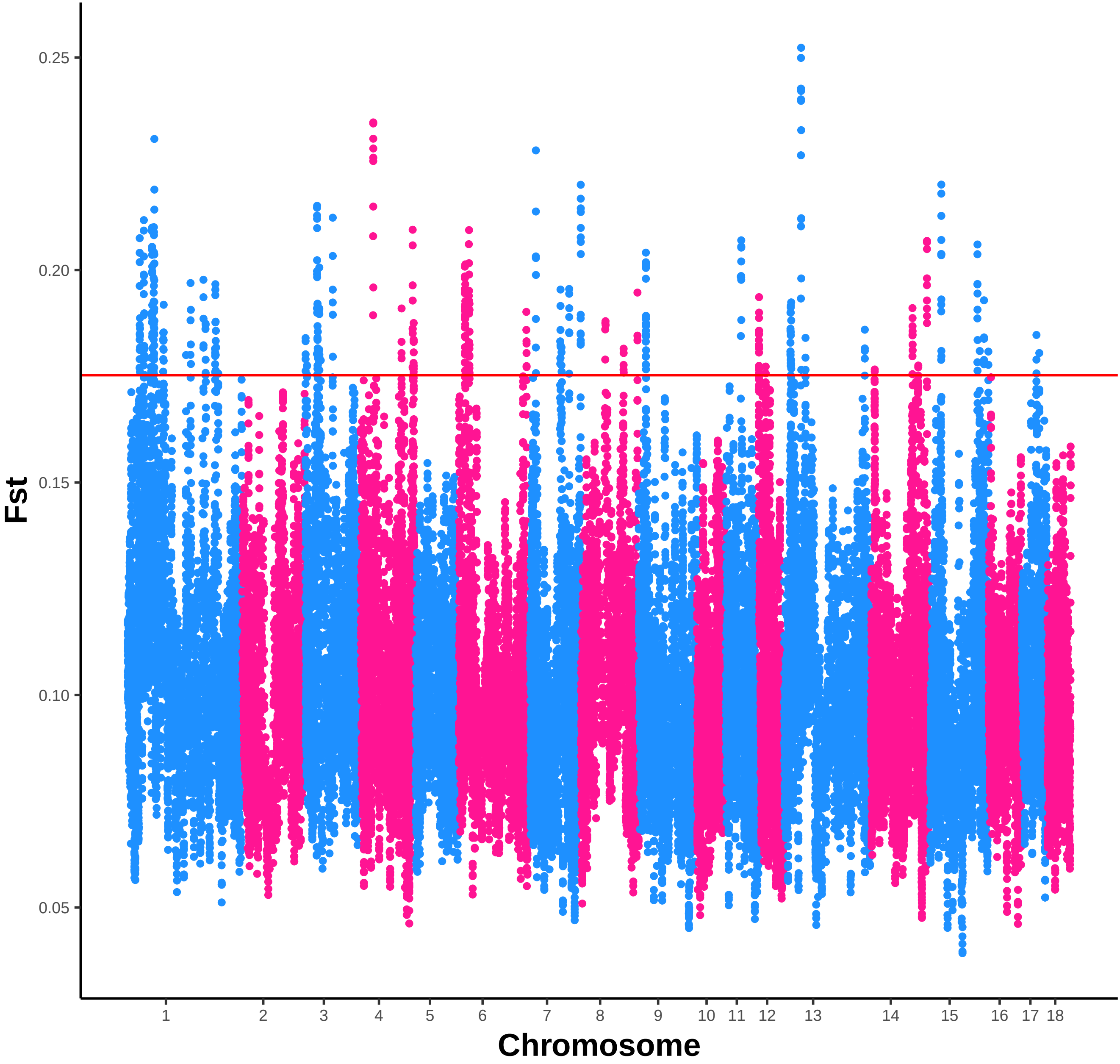

Supplementary Figure 14. Genome wide distribution of FST estimated in sliding windows in Mangalitsa breed.
The continued red line delimits the 99th percentile

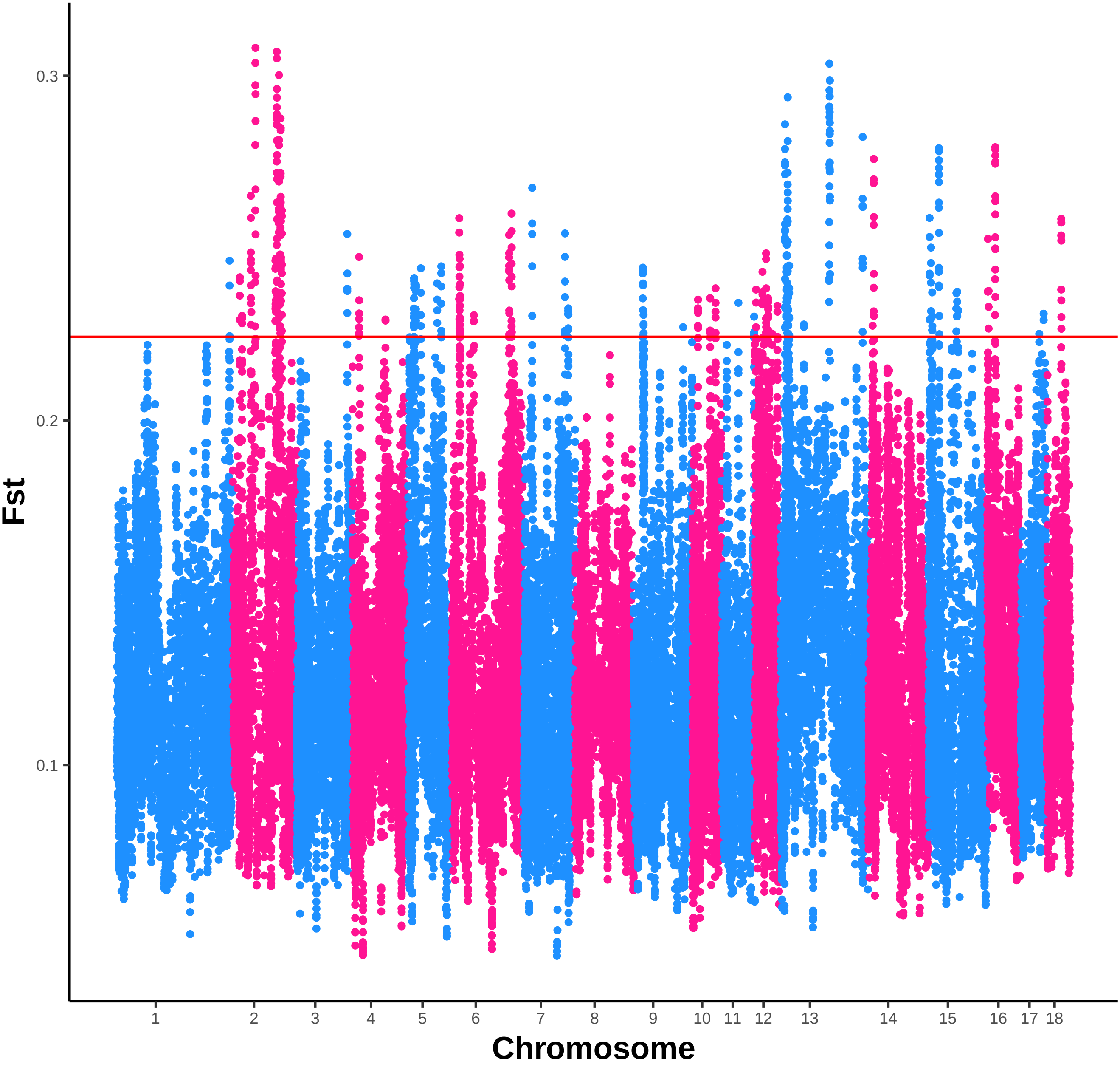

Supplementary Figure 15. Genome wide distribution of FST estimated in sliding windows in Mora Romagnola breed. The continued red line delimits the 99th percentile

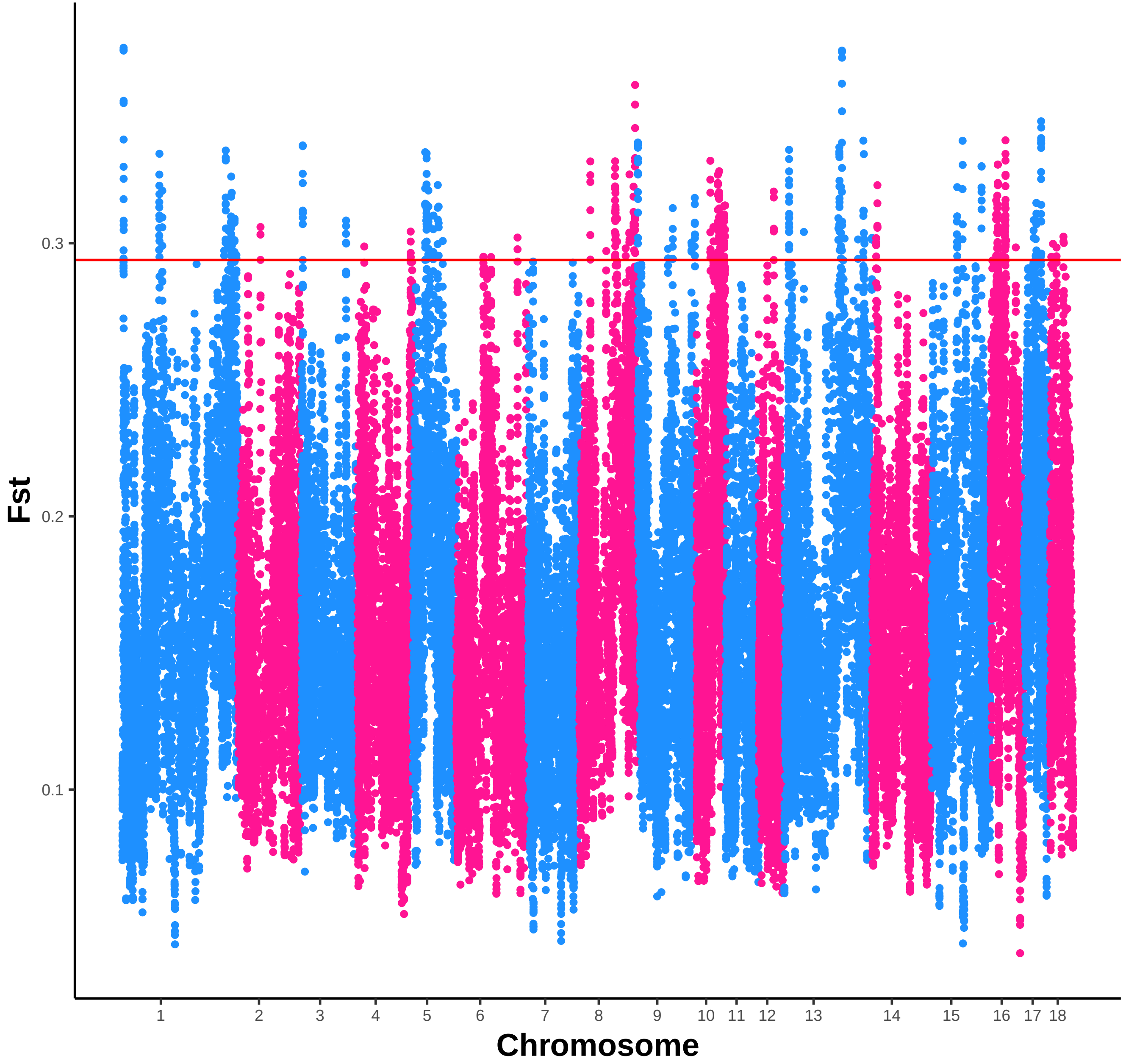

Supplementary Figure 16. Genome wide distribution of FST estimated in sliding windows in Moravka breed. The continued red line delimits the 99th percentile

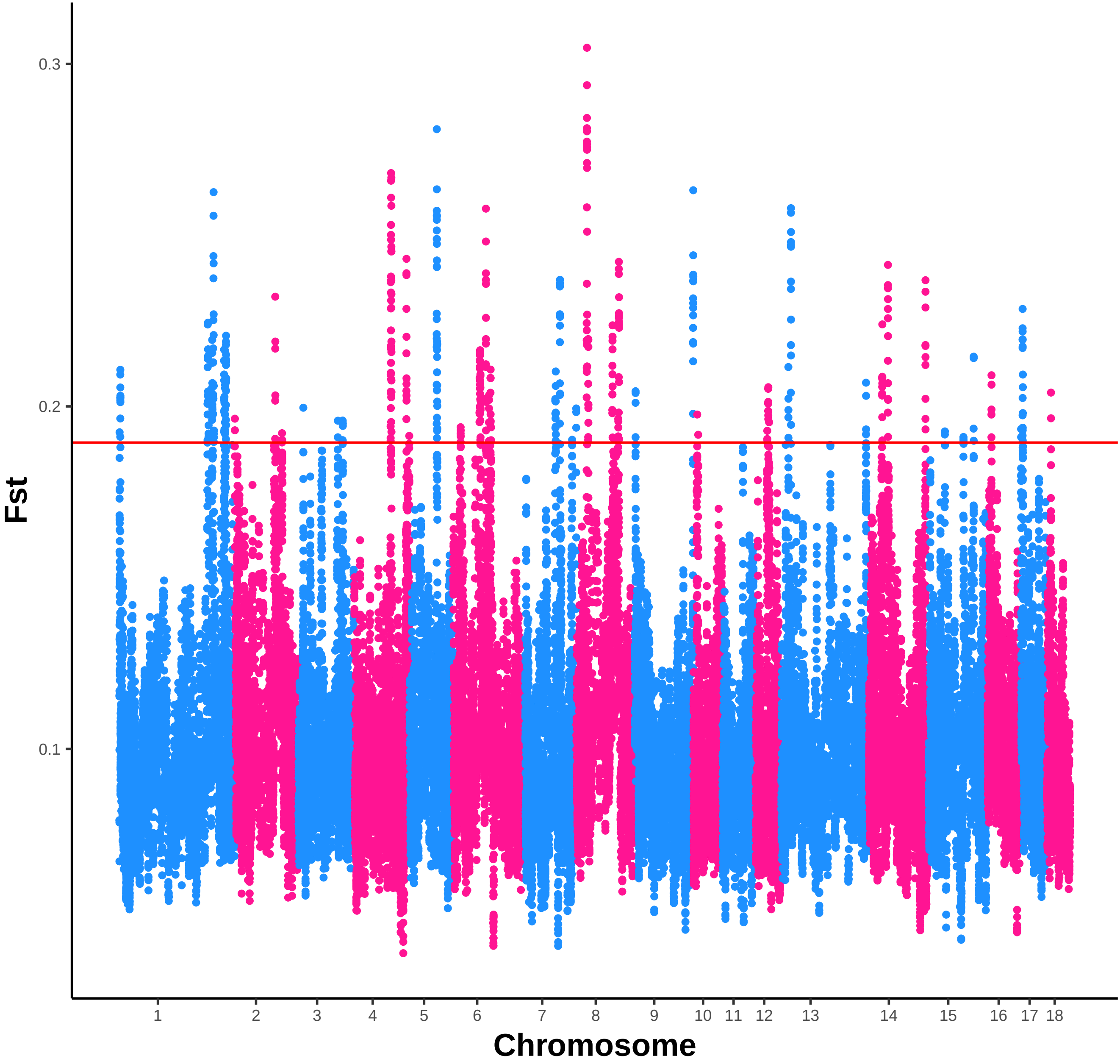

Supplementary Figure 17. Genome wide distribution of FST estimated in sliding windows in Nero Siciliano breed. The continued red line delimits the 99th percentile

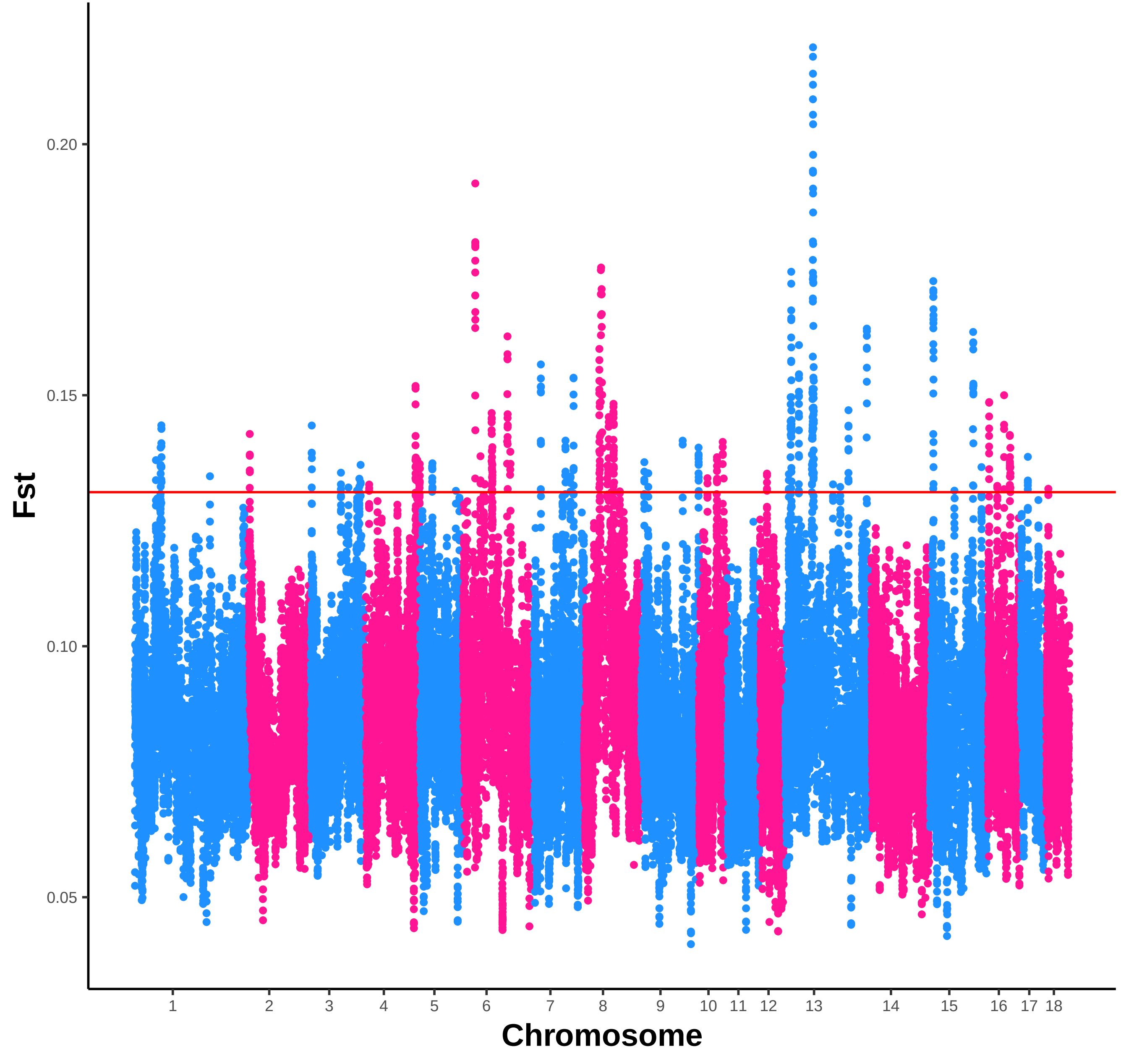

Supplementary Figure 18. Genome wide distribution of FST estimated in sliding windows in Old Type Lithuanian White breed. The continued red line delimits the 99th percentile

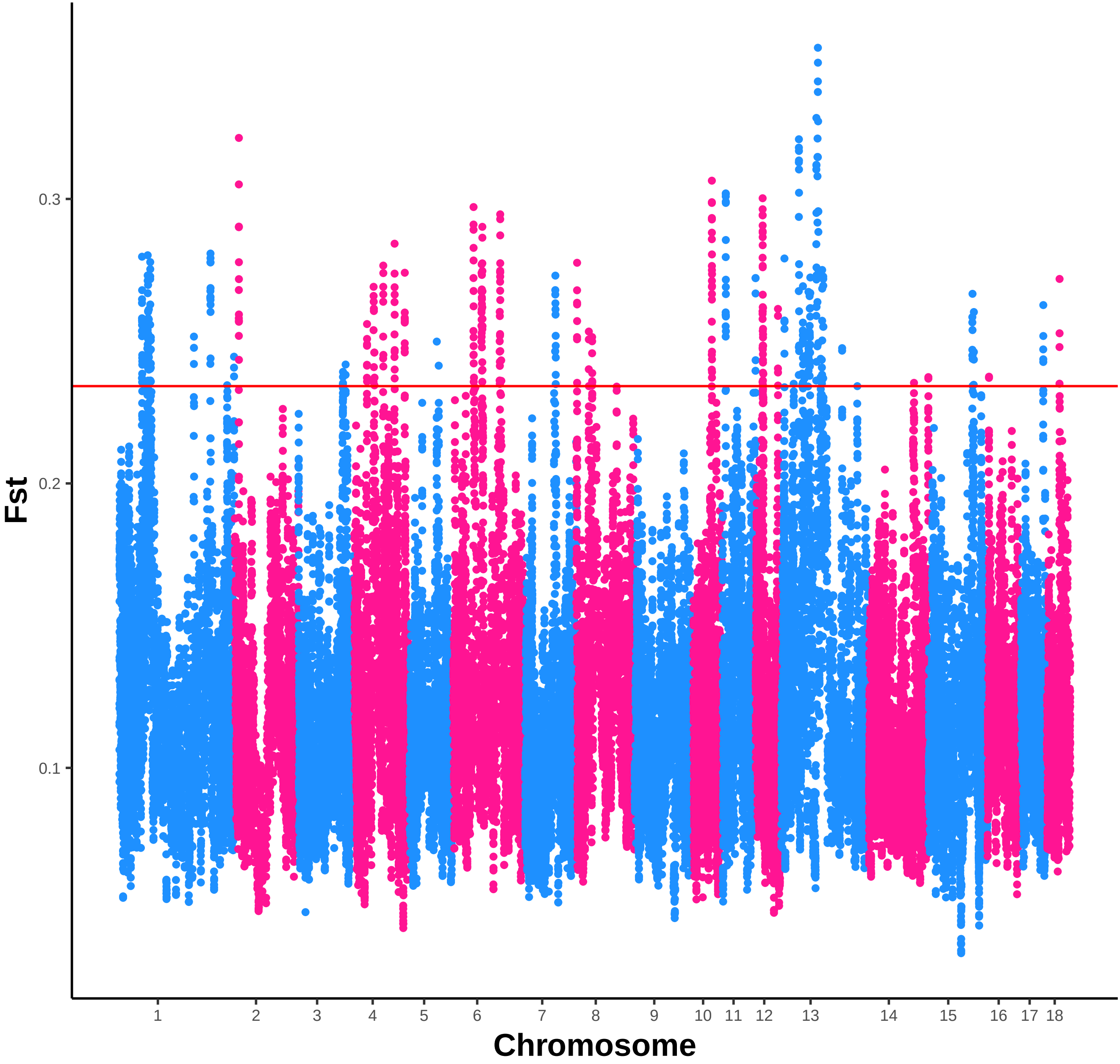

Supplementary Figure 19. Genome wide distribution of FST estimated in sliding windows in Sarda breed.
The continued red line delimits the 99th percentile

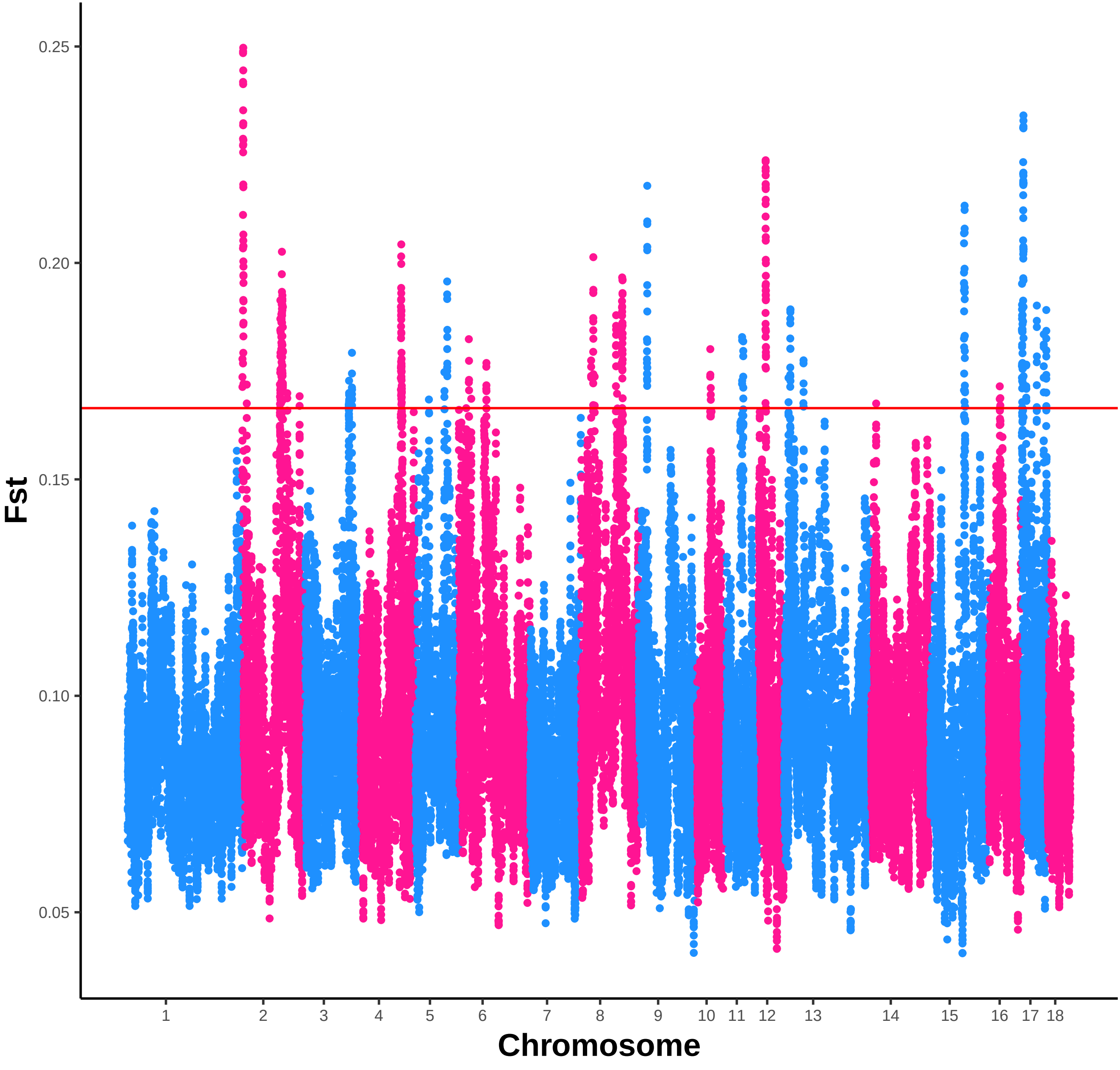

Supplementary Figure 20. Genome wide distribution of FST estimated in sliding windows in Schwaebisch−Haellisches Schwein breed. The continued red line delimits the 99th percentile

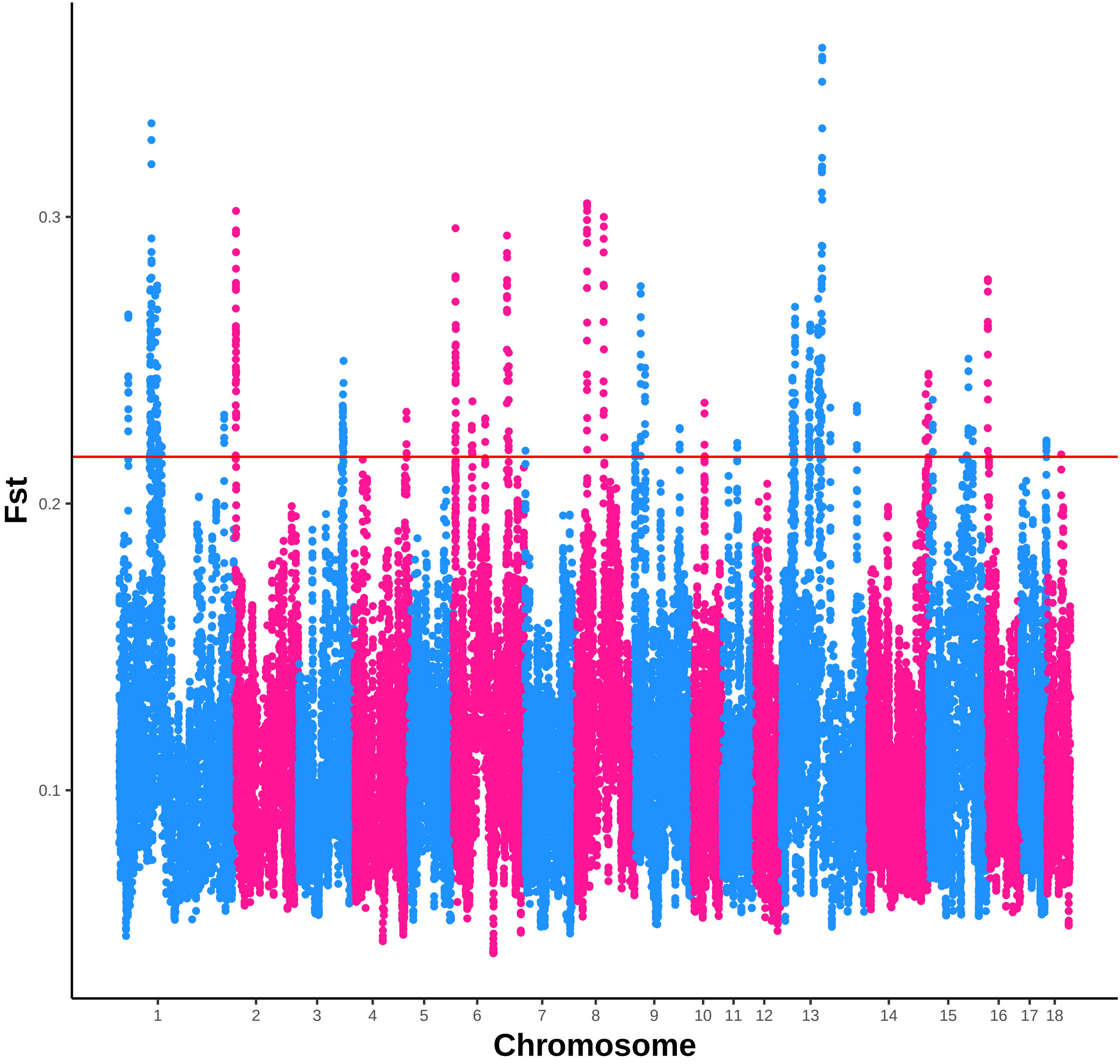

Supplementary Figure 21. Genome wide distribution of FST estimated in sliding windows in Turopolje breed. The continued red line delimits the 99th percentile

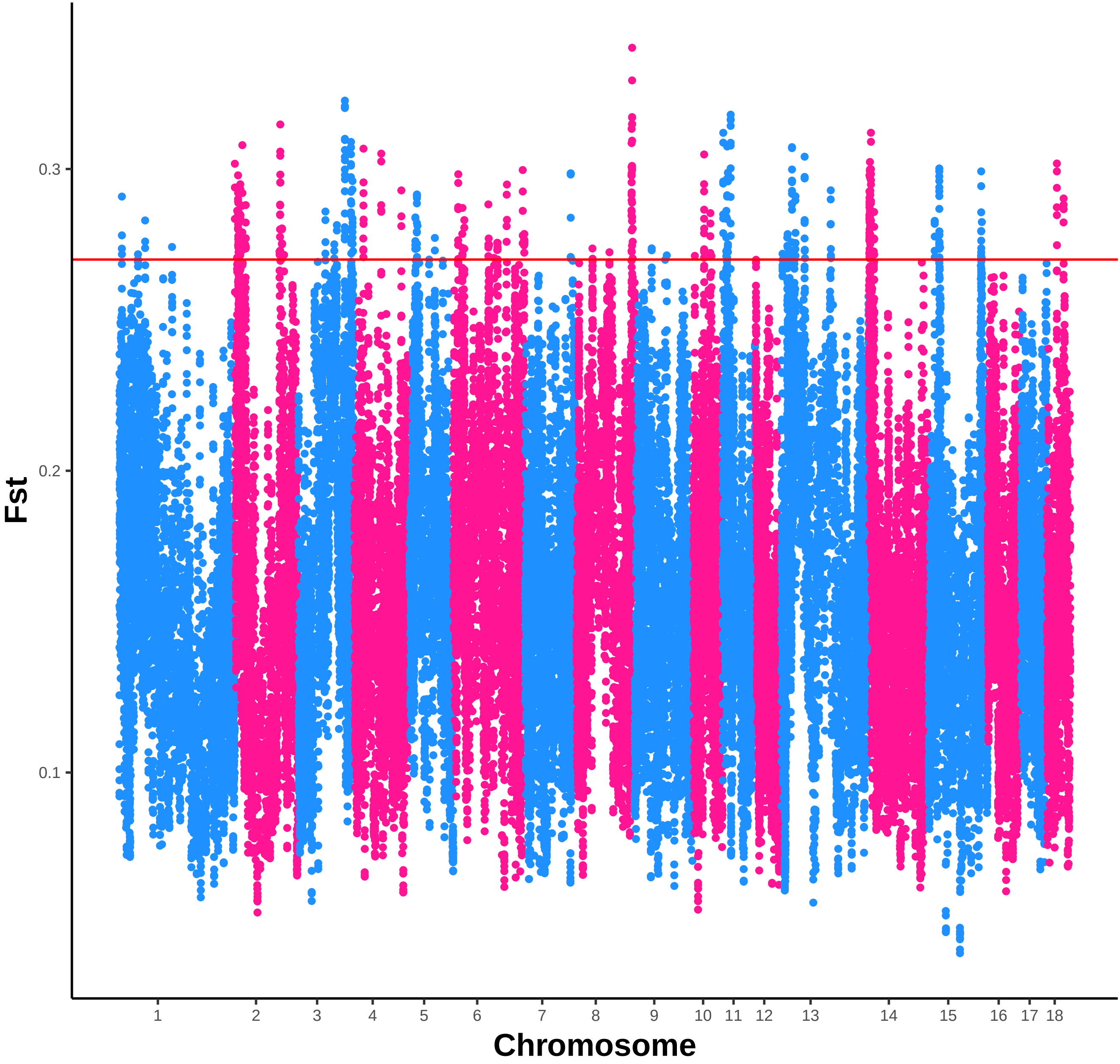

Supplementary Figure 22. Genome wide distribution of FST estimated in sliding windows in Wild Boar breed. The continued red line delimits the 99th percentile

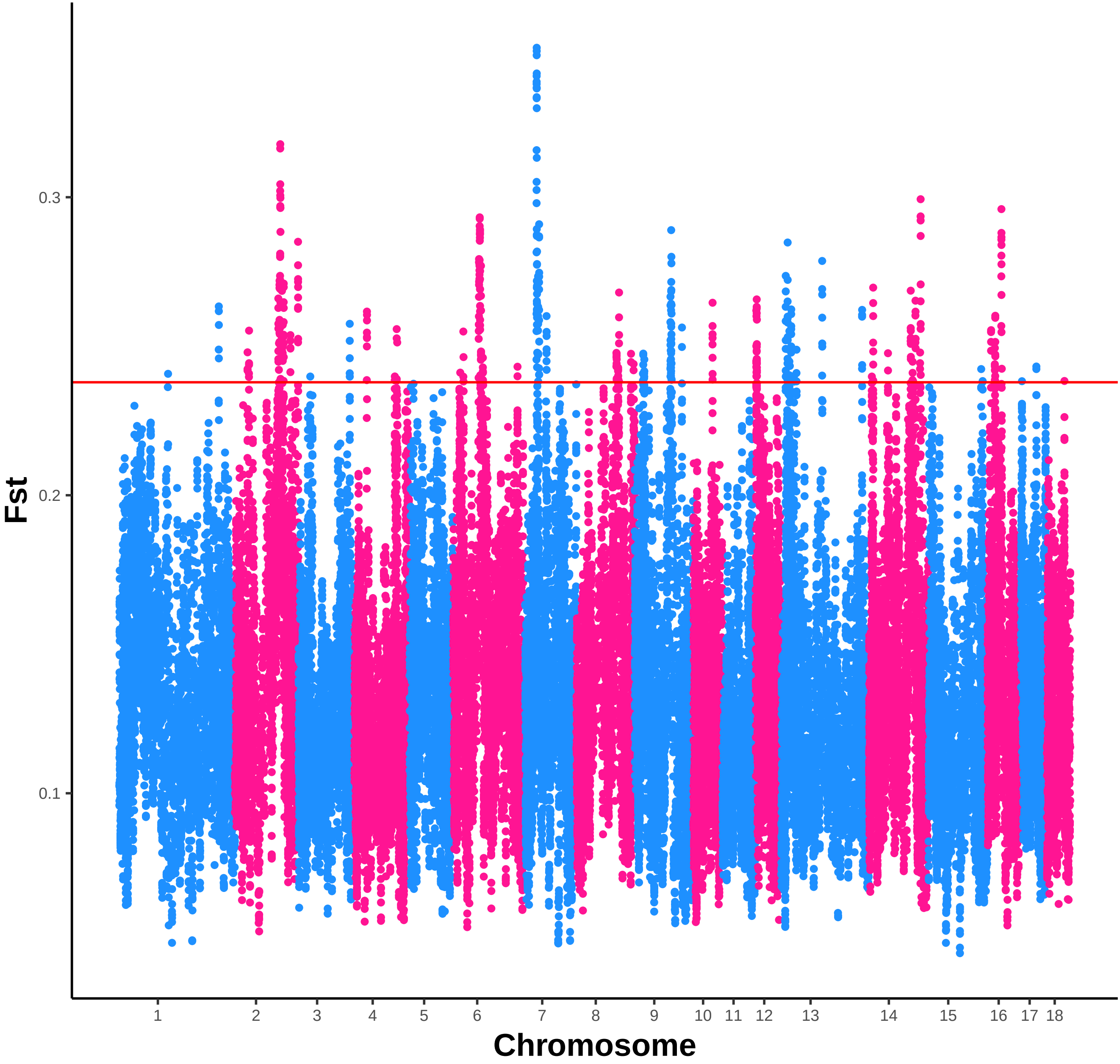

Supplement: Supplementary file 2 — Supplementary material 2 [file 41598_2019_49830_MOESM2_ESM.pdf]
