# Supplementary material for: Genomic diversity, linkage disequilibrium and selection signatures in European local pig breeds assessed with a high density SNP chip
Source: Sci Rep. 2019 Sep 19;9:13546. doi: 10.1038/s41598-019-49830-6 (PMC6753209; doi:10.1038/s41598-019-49830-6)
Supplement: Supplementary file 3 — Supplementary material 3 [file 41598_2019_49830_MOESM3_ESM.pdf]

Genomic diversity, linkage disequilibrium and selection signatures in European local pig breeds assessed with a high density SNP chip

Muñoz M, Bozzi R, García-Casco J, Núñez Y, Ribani A, Franci O, García F, Škrlep M, Schiavo G, Bovo S, Utzeri VJ, Charneca R, Martins JM, Quintanilla R, Tibau J, Margeta V, Djurkin-Kušec I, Mercat MJ, Riquet J, Estellé J, Zimmer C, Razmaite V, Araujo JP, Radović Č, Savić R, Karolyi D, Gallo M, Čandek-Potokar M, Fernández AI, Fontanesi L, Óvilo C

Supplementary Table 1. Minimum allele frequency (MAF) ranges computed for each SNP by breed.

	**MAF range values**									
Breeds	0.01-0.05	0.05-0.10	0.10-0.15	0.15-0.20	0.20-0.25	0.25-0.30	0.30-0.35	0.35-0.40	0.40-0.45	0.45-0.50
Alentejana	18244	5799	4522	4426	4660	3757	4750	5049	4934	4310
Apulo Calabrese	10782	6265	5159	6400	4771	4537	5966	5169	5136	6266
Basque	20428	6186	5577	4785	4357	4194	3801	3959	3580	3584
Bísara	4129	4830	5257	5934	6339	6615	6988	7030	7049	6280
Black Majorcan	12374	7783	5741	5274	5069	4078	5092	5267	5095	4678
Black Slavonian	5343	5019	5382	5992	6057	6291	6468	6742	6965	6192
Casertana	8182	4818	5595	5497	6417	5599	6173	6433	5598	6139
Cinta Senese	8376	7955	8017	5990	6141	4516	4560	5233	4618	5045
Gascon	12187	5100	5576	5328	5183	4344	6206	5918	5532	5077
Iberian	16253	6189	4580	4567	4672	4704	4848	4872	5132	4634
Krškopolje	4917	4163	4981	5502	6195	6397	6808	7387	7595	6506
Lithuanian indigenous wattle	8724	4566	5135	5731	5657	4924	6239	6659	6699	6117
Swallow-Bellied Mangalitsa	19084	4918	4786	4540	4536	4579	4777	4673	4414	4144
Mora Romagnola	22287	5509	6263	4934	4217	2712	3803	3723	3676	3327
Moravka	5222	4850	5733	5757	6062	6247	6836	6734	6930	6080
Nero Siciliano	3600	5058	5854	6420	6332	6500	6562	6821	6937	6367
Old type Lithuanian White	6759	4687	5085	5631	5937	5179	6875	7041	6929	6328
Sarda	1538	3499	4989	5929	6719	6788	7722	7909	8068	7290
Schwäbisch-Hällisches Schwein	6715	4552	4857	5315	5783	6305	6392	6651	7031	6850
Turopolje	29740	4808	4619	4418	3142	3344	2989	2521	2439	2431
Wild Boar	18229	6325	5938	233	6172	6405	174	6718	6834	3422

Supplementary Table 2. Genetic distances among the 21 breeds estimated using Nei's formula (1972).

	AL	AP	BA	BI	BS	CA	CS	GA	IB	KR	LIW	MB	MA	MR	MV	NS	OLW	SA	SW	TU
AP	0.458																			
BA	0.413	0.552																		
BI	0.386	0.508	0.475																	
BS	0.360	0.490	0.455	0.419																
CA	0.420	0.543	0.515	0.474	0.452															
CS	0.344	0.477	0.444	0.409	0.375	0.442														
GA	0.406	0.536	0.462	0.457	0.437	0.496	0.429													
IB	0.276	0.461	0.420	0.391	0.364	0.423	0.348	0.411												
KR	0.396	0.513	0.482	0.439	0.418	0.478	0.414	0.462	0.398											
LIW	0.397	0.512	0.484	0.435	0.421	0.477	0.415	0.460	0.402	0.444										
MB	0.312	0.462	0.421	0.391	0.365	0.422	0.350	0.409	0.316	0.397	0.400									
MA	0.331	0.481	0.440	0.413	0.376	0.443	0.369	0.429	0.336	0.417	0.421	0.341								
MR	0.474	0.604	0.575	0.532	0.509	0.561	0.491	0.555	0.477	0.530	0.535	0.481	0.500							
MV	0.366	0.493	0.456	0.418	0.393	0.455	0.390	0.437	0.371	0.417	0.422	0.370	0.377	0.515						
NS	0.332	0.466	0.431	0.395	0.374	0.429	0.362	0.414	0.337	0.400	0.400	0.336	0.357	0.487	0.374					
OLW	0.448	0.551	0.525	0.474	0.467	0.519	0.461	0.499	0.453	0.482	0.450	0.448	0.469	0.581	0.464	0.441				
SA	0.366	0.489	0.458	0.417	0.397	0.454	0.387	0.438	0.370	0.419	0.418	0.369	0.389	0.510	0.398	0.372	0.456			
SW	0.409	0.522	0.489	0.450	0.432	0.490	0.429	0.473	0.413	0.437	0.452	0.409	0.431	0.551	0.427	0.411	0.489	0.432		
TU	0.389	0.533	0.493	0.462	0.421	0.496	0.421	0.478	0.392	0.466	0.470	0.396	0.409	0.552	0.437	0.410	0.517	0.440	0.480	
WB	0.369	0.526	0.484	0.457	0.428	0.488	0.413	0.474	0.372	0.464	0.467	0.381	0.396	0.543	0.434	0.400	0.518	0.434	0.476	0.455

AL: Alentejana; : Apulo Calabrese; BA: Basque; BI: Bísara; BR: Black Slavonian; CA: Casertana; CS: Cinta Senese; GA: Gascon; IB: Iberian; KR: Krškopolje; LI: Lithuanian indigenous wattle; MB: Majorcan Black; MA: Swallow-Bellied Mangalitsa ; MR: Mora Romagnola; MV: Moravka; NS: Nero Siciliano; OW: Old type Lithuanian White; SA: Sarda; SW: Schwäbisch-Hällisches Schwein; TU: Turopolje; WB: Wild Boar.

Supplementary Table 3. SNPs used in LD decay analyses by population.

Population	SNP
Alentejana	35,706
Apulo Calabrese	41,909
Basque	33,987
Bísara	47,388
Black Slavonian	46,579
Casertana	43,761
Cinta Senese	43,915
Gascon	40,828
Iberian	37,431
Krškopolje	47,561
Lithuanian indigenous wattle	43,862
Majorcan Black	40,666
Swallow-Bellied Mangalitsa	35,980
Mora Romagnola	32,118
Moravka	47,381
Nero Siciliano	48,587
Old type Lithuanian White	45,461
Sarda	49,464
Schwäbisch-Hällisches Schwein	45,506
Turopolje	27,339
Wild Boar	35,228
